# Recyclable, Biobased Polycarbonates and Polyesters by Naphthoxy‐Imine Zinc and Magnesium Complexes

**DOI:** 10.1002/chem.202501271

**Published:** 2025-05-27

**Authors:** Federica Tufano, Maria Vittoria Galotto, Alfredo D'Elia, Federica Santulli, Mina Mazzeo, Marina Lamberti

**Affiliations:** ^1^ Department of Chemistry and Biology “Adolfo Zambelli” University of Salerno via Giovanni Paolo II, 132 Fisciano SA 84084 Italy; ^2^ CIRCC Interuniversity Consortium Chemical Reactivity and Catalysis Bari BA 70126 Italy

**Keywords:** CO_2_ fixation, chemical recycling, polylactide, polytrimethylene carbonate, renewable resources

## Abstract

Naphthoxy‐imine pyridine zinc and magnesium complexes were synthesized and fully characterized by nuclear magnetic resonance (NMR). In the presence of an alcohol as initiator, both complexes promoted the ring‐opening polymerization (ROP) of L‐lactide (L‐LA), ε‐caprolactone (ε‐CL), β‐butyrolactone (β‐BL), trimethylene carbonate (TMC), and 1‐methyl trimethylene carbonate (Me‐TMC), which was purposely synthesized from CO_2_ and the appropriate diol. The zinc complex exhibited notably high activity, particularly in the polymerization of lactide and TMC, and was subsequently employed in the synthesis of polytrimethylene carbonate‐based diblock and random copolymers with both ε‐CL and L‐LA. Furthermore, the zinc complex demonstrated its ability to close the life cycle of the synthesized materials by successfully depolymerizing both polytrimethylene carbonate (PTMC) and its copolymers.

## Graphical abstract

Zinc and magnesium complexes, like skilled composers, craft polymeric symphonies by orchestrating diverse combinations of monomers. Once their performance is complete, the polymers gracefully can be decomposed back into their original monomers, ready to be composed into new symphonies.

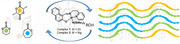

## 1 Introduction

Climate change, along with the environmental challenges of the modern era, often referred to by scientists as the Anthropocene, demands a united commitment toward a more sustainable future. In the realm of plastic materials, this shift emphasizes an increased focus on biopolymers.^[^ ^1^, ^2^ ^]^ Aliphatic polyesters^[^ ^3^, ^4^, ^5^ ^]^ and aliphatic polycarbonates^[^ ^6^, ^7^ ^]^ are exemplary members of this category, as they can be sourced from renewable materials,^[^ ^8^ ^]^ are biodegradable, and offer the advantage of chemical recyclability.^[^ ^9^ ^]^ These features make them particularly appealing for high‐volume applications, such as packaging, while their excellent biocompatibility enables their use in the biomedical field.^[^ ^10^ ^]^ Moreover, their copolymers offer the flexibility to tailor chemical and physical properties while maintaining biocompatibility, thereby broadening the range of potential applications.

Ring‐Opening Polymerization (ROP) of lactones and cyclic carbonates, catalyzed by metal‐based catalysts,^[^ ^2^ ^]^ enables the efficient, controlled, and environmentally friendly production of the aforementioned polymers. Meanwhile, Ring‐Opening COPolymerization (ROCOP) of the same monomers allows for the synthesis of the corresponding copolymers.

Homopolymers of trimethylene carbonate (TMC) have shown excellent histocompatibility, attributed to their neutral degradation and typically exhibit longer degradation times compared to polyesters.^[^ ^11^ ^]^ However, polytrimethylene carbonate (PTMC), being a rubbery polymer, lacks the dimensional stability, and mechanical properties required for use as an implant material. Thus, incorporating esters units, such as ε‐caprolactone^[^ ^12^, ^13^, ^14^ ^]^ or lactide,^[^ ^15^, ^16^, ^17^ ^]^ into PTMC offers numerous advantages. This modification enhances PTMC mechanical properties, including tensile strength and elasticity. Additionally, the degradation rate of the copolymers can be precisely adjusted by varying the ratio of PTMC to ester units, enabling tailored degradation profiles to meet specific biomedical needs. Furthermore, these copolymers can be processed into diverse forms, such as films, fibers, and scaffolds, which broadens their potential applications across fields like biomedicine, packaging, and agriculture.^[^ ^7^ ^]^

Among the numerous catalysts described in the literature, zinc and magnesium complexes^[^ ^18^ ^]^ stand out for their minimal or nontoxic nature, high activity, and remarkable ability to maintain precise control over polymerization processes, even under the most demanding industrial conditions.^[^ ^19^ ^]^ The high versatility of amino‐ and imino‐phenolate ligands made them a common choice as ancillary ligands for both magnesium and zinc, resulting in efficient catalysts in the ROP of cyclic esters^[^ ^20^, ^21^, ^22^, ^23^, ^24^, ^25^ ^]^ and cyclic carbonates.^[^ ^26^, ^27^, ^28^, ^29^, ^30^ ^]^

Recently, some of us have developed zinc and magnesium complexes bearing phenoxy‐imino‐pyridinic ligands that are easy to synthesize, cost‐effective, and exhibit outstanding activity both in the production of polylactic acid (PLA) and in its chemical recycling.^[^ ^31^, ^32^, ^33^, ^34^ ^]^ These complexes were found to be competitive even with the most active zinc catalysts reported in the literature for polymerizations carried out under industrial conditions with nonpurified lactide.^[^ ^35^, ^36^, ^37^, ^38^, ^39^, ^40^ ^]^

Having verified that the substituents on the ligand play a significant role on the catalytic activity and well aware that even the organic ligand can be toxic, in this work we decided to move to a naphthoxy‐imino‐pyridinic ligand, as coordinative environment for both zinc and magnesium. This ligand, simple to synthesize and inexpensive, has also been tested by MTT assays showing no‐cytotoxicity at low concentrations.^[^ ^41^ ^]^

Here, we report the synthesis and characterization of new heteroleptic zinc and magnesium complexes with the naphthoxy‐imino‐pyridinic ligand, and the expansion of the scope of this class of complexes, as catalysts for the homo‐ and copolymerization of various bioderived cyclic monomers (both esters and carbonates) and their copolymers. The performance of the zinc complex was also evaluated in the depolymerization of PTMC and its copolymers, utilizing microwaves as a more sustainable alternative to conventional heating methods.

## 2 Results and Discussion

### 2.1 Synthesis of the Complexes

The proligand was synthesized using a procedure already reported in the literature^,[^ ^41^ ^]^ by reacting 2‐hydroxy‐1‐naphthaldehyde with 2,2‐pyridyl ethylamine under reflux in ethanol, and characterized by NMR (Figures S1, S2).

**Scheme 1:**
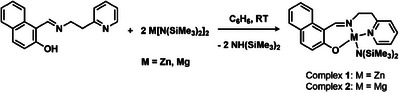
Synthesis of complexes **1** and **2**.

Subsequently, the zinc and magnesium complexes (**1** and **2**, respectively) were obtained by reacting the proligand with an equivalent of the proper metal precursor, M[N(SiMe₃)₂]₂ (M = Zn, Mg), in benzene (Scheme 1). After washing the complexes with hexane to remove the amine produced during the synthesis and drying them in vacuum, they were fully characterized via NMR analysis (Figures S3–S6 for complex **1** and S7–S13 for complex **2**).

The ^1^H NMR spectrum of complex **1** (Figure S3) is consistent with the formation of the tetracoordinated zinc complex shown in the Scheme 1. In particular, the presence of broad signals corresponding to the methylene protons is indicative of the coordination of the pyridinic nitrogen atom to the zinc atom and some fluxionality of the six‐membered metallacycle that forms. The signal at low chemical shift corresponds to the methyl protons of the coordinated trimethylsilyl amido. Further confirmation of the proposed coordinative environment for the zinc center comes from the nuclear overhauser effect correlation between the alpha proton of the pyridinic ring and the proton signal corresponding to the methyl protons of the amido group (Figure S5).

The characterization of the magnesium complex **2** gave similar results to what observed for complex **1**, however both the signals present in the aromatic region of the proton spectrum and the methylene signals were broader, reasonably indicating a greater fluxionality for the magnesium complex with respect to the zinc complex. Variable temperature ^1^H NMR spectra (Figure S10) showed that at −40 °C the fluxional equilibrium becomes slower than NMR acquisition time and an AA'BB’ pattern can be appreciated for methylene protons of the ligand.

### 2.2 ROP of Lactones and Cyclic Carbonates

**Scheme 2:**
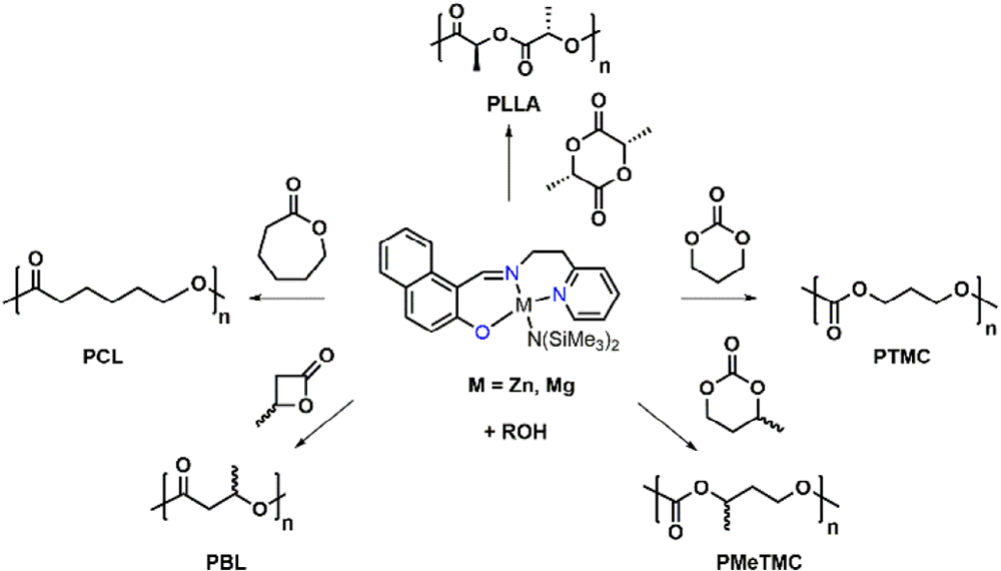
ROP of lactones and cyclic carbonates promoted by complexes **1** and **2**.

The catalytic activities of complexes **1** and **2** for the ROP of different lactones and cyclic carbonates (Scheme 2) were assessed under different reaction conditions, and the polymerization results are summarized in Table 1.

**Table 1:** ROP of cyclic esters and carbonates by complexes **1** and **2**.

Entry^[^ ^a^ ^]^	Complex	Monomer [equiv]	Initiator [equiv]	Temp [°C]	Solvent (volume)	Time	Conv^[^ ^b^ ^]^ [%]	*M*_n_^th[^ ^c^ ^]^ [kDa]	*M*_n_^GPC[^ ^d^ ^]^ [kDa]	Ɖ
1	**1**	L‐LA (100)	^i^PrOH	20	DCM (2 mL)	1 minute	90	13.0	14.7	1.1
2	**1**	L‐LA (5000)	BnOH (50)	150	‐	5 minutes	88	12.7	13.6	1.1
3	**2**	L‐LA (100)	^i^PrOH	20	DCM (2 mL)	1.5 hours	80	11.5	11.5	1.3
4	**1**	β‐BL (100)	^i^PrOH	70	Toluene (2 mL)	6 hours	62	5.3	*4.9*	
5	**2**	β‐BL (100)	^i^PrOH	70	Toluene (2 mL)	6 hours	< 1	‐	‐	
6	**1**	β‐BL (100)	^i^PrOH	70	‐	30 minutes	98	8.4	8.5	1.4
7	**2**	β‐BL (100)	^i^PrOH	70	‐	2 hours	56	4.8	*3.9*	
8	**1**	ε‐CL (100)	^i^PrOH	20	DCM (2 mL)	1.5 hours	85	9.7	8.7	1.3
9	**2**	ε‐CL (100)	^i^PrOH	20	DCM (2 mL)	45 minutes	90	10.3	8.6	1.4
10	**1**	TMC (200)	BnOH	20	DCM (0.4 mL)	1 minute 5 minutes	85 89	18.1	16.3	1.6
11	**2**	TMC (200)	BnOH	20	DCM (0.4 mL)	1 minute 5 minutes	82 95	19.4	20.3	1.7
12	**1**	TMC (200)	BnOH	20	dioxolane (0.4 mL)	30 minutes 1.5 hours	52 87	17.8	11.8	2.1
13	**2**	TMC (200)	BnOH	20	dioxolane (0.4 mL)	30 minutes 1.5 hours	73 87	16.7	16.5	1.7
14	**1**	TMC (500)	BnOH (5)	70	‐	10 minutes	100	10.2	19.2	2.0
15	**2**	TMC (500)	BnOH (5)	70	‐	10 minutes	97	9.9	6.0	1.8
16	**2**	TMC (500)	BnOH	70	‐	1 hours	98	50.0	52.2	2.2
17	**1**	Me‐TMC (50)	BnOH	20	‐	30 minutes	72	4.2	*3.1*	
18	**2**	Me‐TMC (50)	BnOH	20	‐	1 hour	70	4.1	*5.2*	

^[a]^ General conditions: metal complex = 4 µmol for cyclic carbonates, 5 µmol for cyclic esters; one equivalent of alcohol, except when differently specified.

^[b]^ Determined by ^1^H NMR spectral data.

^[c]^ *M*_n_^th^ (kDa) = MM_monomer_ × ([monomer]_0_/[Cat]_0_) × monomer conversion)/10^3^ Da/kDa.

^[d]^ Experimental *M*_n_ and *M*_w_/*M*_n_ (Đ) values were determined by SEC in THF using polystyrene standards and corrected using a factor of 0.58 for PLA, 0.54 for PBL, 0.56 for PCL, and 0.73 for PTMC with theoretical mass in the range 5–10 kDa and 0.88 for PTMC with theoretical mass > 10 kDa. Experimental *M*_n_ indicated in italic have been determined by matrix assisted laser desorption/ionization (MALDI) analysis.

We chose conditions for the ROP of lactide in which zinc complexes bearing the analogous class of ligands (phenoxy‐imino pyridines) have already been explored. In this way we verified that the zinc complex with naphthoxy‐imino pyridine ligand has a higher activity, with a TOF (9800 hour^−1^ for entry 1, Table 1, after 30 seconds) approximately double compared to the highest obtained previously (5050 hour^−1^ after 30 seconds).^[^ ^31^ ^]^ Complex **1** showed very high activity also under industrial conditions, that is, working in unpurified molten L‐lactide (L‐LA) (5000 eq), at 150 °C, and with a high amount of BnOH (50 eq). The high TOF (52 800 hour^−1^ for entry 2, Table 1) registered in this case, demonstrates the high stability of the complex both to protic impurities that to high temperature.

Then we moved on to compare the behavior of the zinc complex with that of magnesium in the ROP of different monomers.

**Figure 1:**
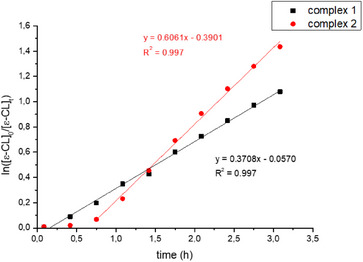
Pseudo first‐order kinetic plots for the consumption of ε‐CL (100 equiv) in CD_2_Cl_2_ at 20 °C by complex **1** (black squares, entry 1 in Table S1) and complex **2** (red circles, entry 2 in Table S1) in combination with ^i^PrOH.

As for the cyclic esters, the zinc complex **1** shows an activity two order of magnitude higher than that of the magnesium complex **2** in the polymerization of L‐LA (compare entries 1 and 3, Table 1), furthermore it is also able to polymerize β‐butyrolactone at 70 °C in toluene (entry 4, Table 1), whereas the magnesium complex was not active under these conditions (entry 5, Table 1). Working in neat monomer, also magnesium manages to promote the ROP of β‐butyrolactone, although the zinc complex remains much more active (entries 6 and 7, Table 1). On the other hand, the magnesium complex showed an activity slightly higher that of zinc in the ROP of ε‐caprolactone (entries 8 and 9, Table 1). For ε‐caprolactone polymerization we also conducted kinetics studies in J‐Young NMR tubes, working in CD_2_Cl_2_ at 25 °C, following the reaction by ^1^H NMR spectra (Figure 1 and Table S1). Magnesium complex **2** showed an induction period of about 0.6 hour, but overall, it was about one and half times faster than zinc complex **1** (*k*_obs_ = 6.06 ⋅ 10^−1^ and 3.71 ⋅ 10^−1^, respectively). The observed induction period suggests that for the magnesium‐based catalytic system the formation of the active species is not immediate. However, in both cases, pseudo first order kinetics with respect to monomer concentration were observed. The higher activity of the zinc complex in the polymerization of lactide compared to the related magnesium complex had already been observed for complexes with phenoxy‐imino pyridine ligands, in a previous work.^[^ ^32^ ^]^ However, the beneficial effect of the naphthoxy‐imino pyridine ligand, reasonably due to its greater propensity to donate electrons to the metal, is highlighted by the increase in activity found for both metals.

Moving to cyclic carbonates, considering that for TMC the monomer‐polymer equilibrium strongly depends on monomer concentration and on the nature of the solvent, we first studied polymerization reactions by varying the concentrations (Table S2). Then, once the best conditions were selected, we carried out the polymerization experiments to compare zinc and magnesium complexes (entries 10–13 in Table 1). In this case, we observe similar high activities in the ROP of TMC, both in solution at 20 °C, that in neat monomer at 70 °C (entries 14 and 15, Table 1). At this temperature, we also prepared PTMC with high‐molecular masses (*ca* 50 kDa, entry 16, Table 1). Noteworthy, both complexes are also active in a green solvent, such as dioxolane (entries 12 and 13, Table 1), although the activities in this solvent were found to be lower than those in dichloromethane.

Subsequently, the activity of the complexes in the ROP of 1‐methyl‐TMC was evaluated in the absence of solvent at 20 °C (entries 17 and 18, Table 1), verifying that, in this case, zinc has an activity approximately double compared to magnesium.

In more details, 1‐methyl trimethylene carbonate (1‐Me‐TMC) was synthesized from biorenewable sources, such as CO_2_ and 1,3‐butanediol.^[^ ^42^, ^43^ ^]^ It is an asymmetric carbonate, therefore the resulting polymer will have a regioregularity that depends on the relative preference of formation of the three possible microstructures: “head‐to‐head” (HH), “head‐to‐tail” (HT), and “tail‐to‐tail” (TT), as shown in Scheme S9. Appropriate integration of the carbonyl region of the ^13^C NMR spectrum allows the determination of the *X*_reg_ (*X*_reg_ = HT/(HT + HH + TT)).^[^ ^44^ ^]^ In our case the magnesium complex showed slightly greater regioselectivity (*X*_reg_ = 0.87, as calculated by integrating the ^13^C NMR showed in Figure S24) than the zinc complex (*X*_reg_ = 0.80). Finally, it should be underlined that the presence of the initiation end groups deriving from primary and secondary insertion (Figure S23), in comparable percentages, allows us to conclude that initiation, unlike propagation, is not regiocontrolled. Similar behaviors have already been observed in the literature for other catalytic systems.^[^ ^29^, ^45^ ^]^

In general, a good agreement has been observed between the theoretical masses and the experimental masses of the obtained polymers, determined through SEC analysis, with slightly higher dispersity values for the polymers obtained with the magnesium complex. These results suggest a good control of the polymerization process, with all monomers, especially by the zinc complex.

The characterization of the polymers performed through ^1^H NMR and MALDI‐ToF analysis shows that in all cases the end groups are those expected on the basis of initiation on the metal‐alkoxide bond and termination by hydrolysis. The most representative spectra are reported in the Supplementary Information (Figures S14–S21 and S23–S25). It's worth noting that the MALDI spectra of all polymers show only one distribution, indicating the absence of collateral reactions, such as transesterifications for polyesters and decarboxylations for polycarbonates. These observations further reinforce the notion that these complexes promote well‐controlled polymerization processes.

### 2.3 ROCOP of Lactones With TMC

ROCOP is a powerful technique for synthesizing copolymers that exhibit enhanced thermal and mechanical properties compared to their homopolymers. Copolymers incorporating carbonate units are particularly promising for a range of biomedical applications due to their increased flexibility, reduced acidity of degradation products, and unique hydrolytic degradation profile. Through rational changes in comonomer ratios, materials with different degradation times,^[^ ^46^, ^47^, ^48^ ^]^ and tunable mechanical properties^[^ ^49^, ^50^ ^]^ have been fabricated.

**Scheme 3:**
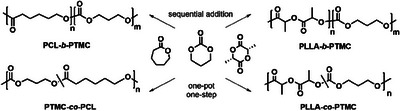
TMC‐based diblock and random copolymers synthesized by complex **1**.

The remarkable activity and versatility of complex **1** prompted us to explore its catalytic performance in the ROCOP of TMC with both ε‐CL and LLA (Scheme 3 and Table 2).

**Table 2:** Sequential and simultaneous copolymerization reactions of TMC with ε‐CL and L‐LA by complex **1**.

Entry^(^ ^a^ ^)^	TMC equiv	Mon2 equiv	Conv^[^ ^b^ ^]^ TMC [%]	Conv^[^ ^b^ ^]^ Mon2 [%]	L_TMC_	L_Mon2_	*M*_n_^th[^ ^c^ ^]^ [kDa]	*M*_n_^exp[^ ^d^ ^]^ [kDa]	Ð^[^ ^d^ ^]^	*T*_g_ [°C]
1	200	LLA 200	90	83	Diblock copolymer		43.3	48.4	2.3	−20.0, 53.2
2	100	ε‐CL 100	74	100	Diblock copolymer		19.0	30.2	1.7	−21.0
3	100 + LLA 100		88	95	2.6	2.6	22.7	25.1	1.7	17.0
4	250 + LLA 250		57	82	2.2	3.8	45.9	‐	‐	29.6
5	250 + ε‐CL 250		86	65	2.6 (2.3)^[^ ^e^ ^]^	1.8 (1.8)^[^ ^e^ ^]^	40.4	47.3	1.7	−42.8
6	500 + ε‐CL 500		96	66	2.5 (2.5)^[^ ^e^ ^]^	1.6 (1.7)^[^ ^e^ ^]^	86.6	53.3	1.8	−36.7

^[a]^ General conditions: Polymerization experiments carried out in bulk conditions. [**1**] = 4 µmol; one equivalent of benzyl alcohol (0.0185 M in THF).

^[b]^ Determined by ^1^H NMR spectral data.

^[c]^ *M*_n_^th^ = (102.03 x [TMC]_0_: [I]_0_ x C_TMC_%) + (144.13 x [L‐LA]_0_: [I]_0_ x C_L‐LA_%) or *M*_n_^th^ = (102.03 x [TMC]_0_: [I]_0_ x C_TMC_%) + (114.14 x [ε‐CL]_0_: [I]_0_ x Cε‐_CL_%).

^[d]^ Experimental *M*_n_ (kDa) and *M*_w_/*M*_n_ (Đ) values were determined by SEC in THF.

^[e]^ Average lengths of carbonate and caproyl blocks in chains for a completely random distribution of units calculated for the experimental molar fraction of TMC in the copolymer.^[^ ^12^ ^]^

First, diblock copolymers were prepared by sequential polymerization. As reported by other authors in the literature, the TMC block fails to form after the polymerization of the lactide, so in this case the cyclic carbonate was polymerized first and then lactide was added (entry 1, Table 2). In the case of ε‐CL the order of addition of the two monomers was irrelevant, and the second monomer was added after the expected time to achieve complete conversion of the first monomer (entry 2, Table 2). Both the masses of the products and the diffusion ordered spectroscopy (DOSY) spectra confirmed the formation of the desired copolymers (Figures S27 and S30). By DSC analysis (Figures S28 and S31), the *T*_g_ of the polycarbonate block was observed around −20 °C for both copolymers, as for the second block both the *T*_g_ (53.2 °C) and the *T*_m_ (169.88 °C) were appreciable for the lactide block,^[^ ^51^ ^]^ while only the *T*_m_ (57.79 °C) was observed for the ε‐caprolactone block.

**Figure 2:**
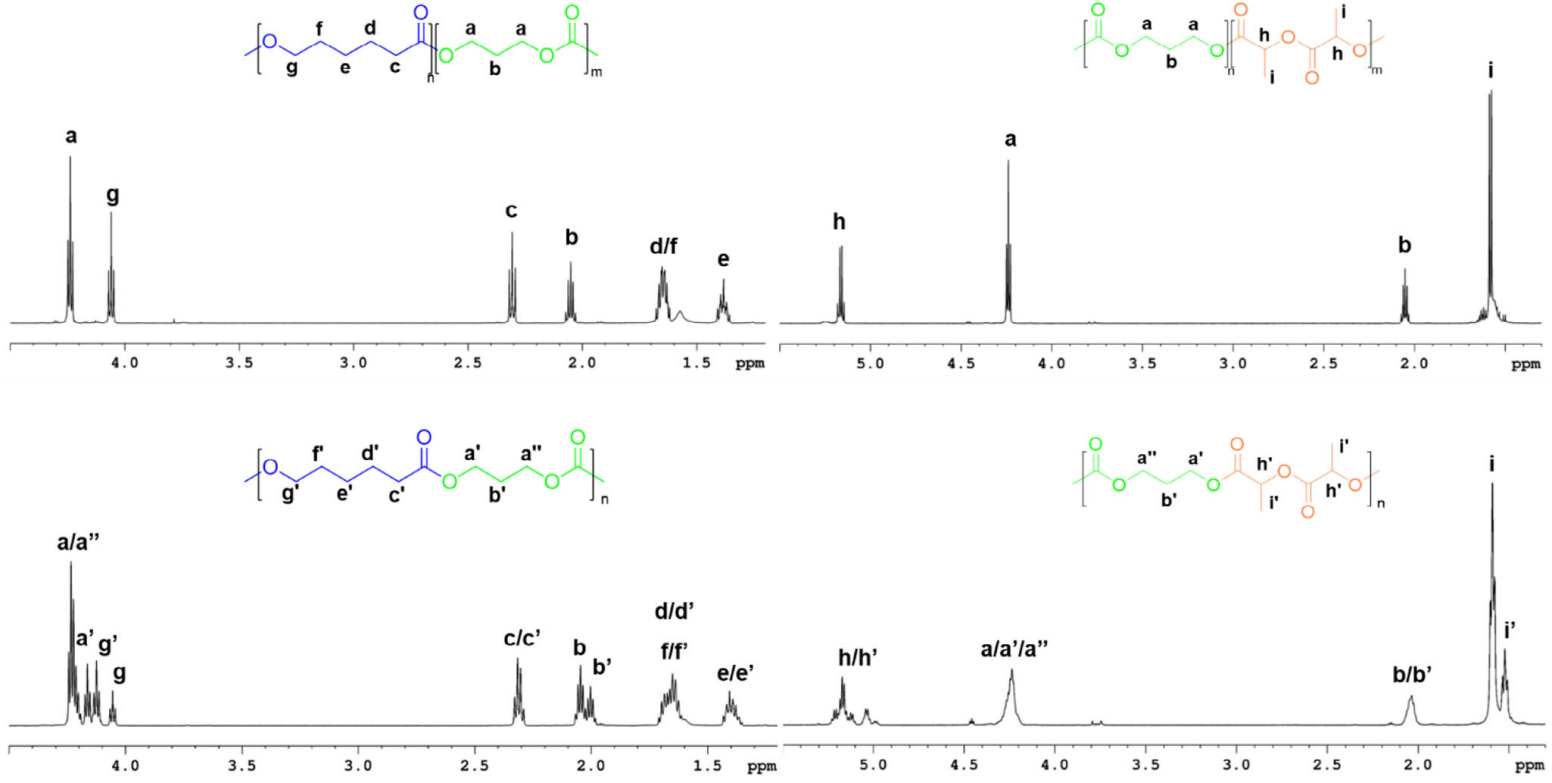
^1^H NMR spectra of the diblock (up) and random (down) copolymers synthesized in this work (600 MHz, CDCl_3_, 298 K).

Then, complex **1** was tested as initiator for simultaneous copolymerization of 1,3‐trimethylene carbonate with L‐LA and ε‐CL, in bulk at 110 °C (Scheme 3). The molecular weights were determined by gel permeation chromatography (GPC) and the composition of the copolymers were determined from the ^1^H NMR spectra by taking the ratio of the peak areas corresponding to the TMC α‐methylene protons at δ = 4.0–4.2 ppm and 1.95–2.10 ppm, for LA monomer methyl protons at δ = 1.4–1.6 ppm and for ε‐CL methylene signals at δ = 2.27–2.35 ppm. The ^1^H NMR spectra in Figure 2 show the signals of both polymer chains in the block copolymers and the additional signals due to heterodyads in the spectra of the random copolymers.

In all cases, the absence of signals around 3.1 ppm in the ^1^H NMR spectra of the copolymers (Figure S32) ruled out the formation of ether units due to decarboxylation of carbonate linkages, even at 110 °C.

At a [TMC]:[LLA]:[**1**]:[BnOH] ratio of 100:100:1:1, TMC and LLA underwent fast copolymerization and approached 88% and 95% conversions, respectively, in 12 minutes (entry 3, Table 2). While working in the same conditions but with 250 equivalents of both monomers, 57% of TMC and 82% of LLA were converted in 10 minutes (entry 4, Table 2). The deviations in copolymer compositions compared to the monomers feed ratios indicate a preference of the catalyst for LLA incorporation. The preferential incorporation of lactide is also corroborated by the observed average block length of lactyl units calculated using standard copolymer equations^[^ ^17^ ^]^ by both ^1^H and ^13^C NMR for the copolymerization at lower conversions (entry 4, Table 2). In the ^13^C NMR spectrum (Figure S33) of copolymer obtained in entry 4, the absence of signals for isolated (TLT) or higher odd number (TLLLT) lactyl units, indicates the absence of transesterification reactions with this catalyst system,^[^ ^49^ ^]^ however small signals have been observed for copolymer of entry 3 and their intensities increase when reactions were conducted beyond the time required for the complete consumption of the monomers.

Subsequently, one‐pot one‐step copolymerizations in [ε‐CL]:[TMC]:[1]:[BnOH] ratio of 250:250:1:1 and 500:500:1:1 were conducted at 110 °C (entries 5 and 6, Table 2). After 5 minutes a conversion of 90% and 60% was calculated for TMC and ε‐CL, respectively; similar conversions were registered for the copolymerization of 500 eq of monomers after the same time. For these copolymerizations a greater propensity of complex **1** to polymerize the TMC is observed compared to the ε‐CL. The experimental average lengths of the carbonate (L_TMC_) and caproyl (L_CL_) units were determined from the carbonyl regions in the ^13^C NMR spectra (see for example Figure S36), according to the equations reported in the literature.^[^ ^12^ ^]^ As expected, the lengths of the TMC blocks are slightly longer than those of CL. However, the calculated block lengths are in all cases close to the values determined for copolymers containing the molar fraction of TMC evaluated by ^1^H NMR, and with a completely random distribution of carbonate and caproyl units.^[^ ^12^ ^]^ Also, worthy of note are the molecular masses of the obtained copolymers that can be adjusted in a satisfactory manner by varying the number of equivalents of the two monomers in the feeds.

The thermal properties of these four copolymers were analyzed by differential scanning calorimetry (DSC). All samples were amorphous and no crystalline phase was found in these copolymers (see for example Figures S34 and S37). The *T*_g_ values (reported in Table 2) are in good agreement with those found in the literature for copolymers of similar compositions and similar masses.^[^ ^12^ ^]^ As expected, these values ​​are intermediate between the *T*_g_’s of the respective homopolymers.

### 2.4 Chemical Recycling to Monomer

Aliphatic polyesters and polycarbonates on which we have focused our attention, are not only derived from renewable sources and biodegradable but are also chemically recyclable. This feature is crucial for supporting the shift from the current linear economy model to a more sustainable circular economy framework.^[^ ^52^, ^53^, ^54^ ^]^

**Scheme 4:**
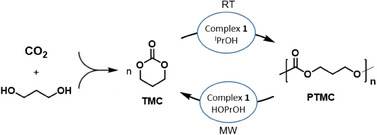
Chemical recycling of polytrimethylenecarbonate (PTMC).

Since in previous works,^[^ ^31^, ^32^, ^34^ ^]^ we showed the high efficiency of this class of zinc complexes in the chemical recycling of polylactide, in this study, we decided to investigate the ability of the synthesized zinc complex in the chemical recycling reactions of PTMC, utilizing microwaves as the heating source. Both the depolymerization of PTMC^[^ ^50^, ^52^ ^]^ and its copolymers to the corresponding monomers were investigated (Scheme 4).

An *ad hoc*‐synthesized PTMC (*M*_n_ = 15 kDa, Ɖ = 1.7) was dissolved in anhydrous acetonitrile. To this solution, the zinc complex (15 mol%) pretreated with an equivalent of alcohol was added. To avoid the formation of multiple species in the product mixtures, propanediol was chosen as the alcohol. The reaction mixture was then heated to 95 °C using microwaves. Satisfactorily, after 2 hours, the polymer was converted to TMC with an 88% yield (entry 1 in Table 3).

**Table 3:** Depolymerization reactions of PTMC by complex **1**.

Entry^[^ ^a^ ^]^	Complex **1** [equiv]	Propandiol [equiv]	Time [h]	Conversion^[^ ^b^ ^]^ [%]
1	0.15	0.15	2	88
2	0.15	0.15	0.5	30
3	0.15	0.15	1	73
4	0.15	0.15	1.5	81
5	0.075	0.075	2	66
6	0.05	0.05	2	11
7	0.15	0.45	2	86
8	0.15	‐	2	<1
9	‐	0.15	2	<1

^[a]^ General conditions: depolymerization experiments were carried out by using 0.1 mmol of PTMC (12 mg), 15 µmol of catalyst (0.15 equiv relative to carbonate linkages) and 15 µmol of propandiol, except when differently specified; solvent: 1 mL of CH_3_CN; temperature: 95 °C by microwave irradiation (20 W power).

^[b]^ Determined by ^1^H NMR spectral data (see for example Figure S38).

The experiments conducted at different times (entries 1–4, Table 3) show that the depolymerization increases linearly over time (Figure S39) until it reaches a plateau, which reasonably corresponds to the monomer/polymer equilibrium under those reaction conditions. Additionally, attempts were made increasing the amount of alcohol (entry 7, Table 3) relative to the zinc complex and decreasing the catalyst loading (entries 5 and 6, Table 3). It was found that increasing the alcohol amount does not improve the catalytic performance, while reducing the catalytic loading to 0.05 equiv significantly compromises the reaction progression.

Additionally, comparative tests were conducted to evaluate the activity of both the alcohol and the zinc complex as single catalytic component (entries 8 and 9, Table 3). In both cases, no conversion was observed after 2 hours under the conditions of entry 1, thus demonstrating that their combined action is necessary to promote PTMC depolymerization.

Then, we evaluated the activity of 1,5,7‐Triazabicyclo[4.4.0]dec‐5‐ene (TBD), an organocatalyst previously reported^[^ ^30^ ^]^ for this type of reaction: under the same conditions as entry 1, TBD (0.15 quiv) showed a significantly lower conversion (35% in 2 hours).

**Scheme 5:**
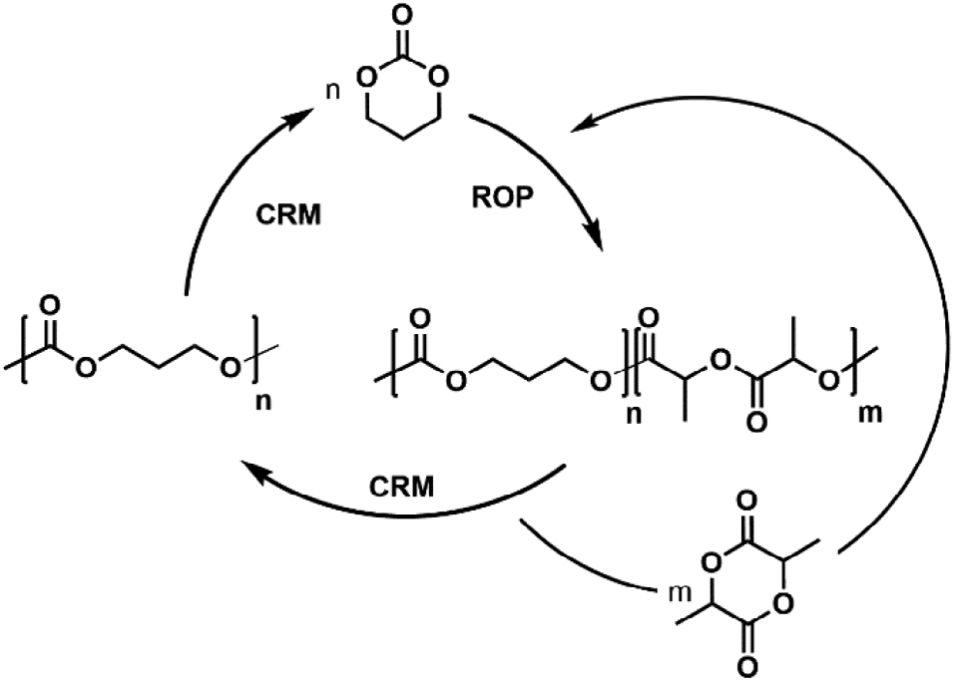
Closed loop for the synthesis of block copolymers PLLA‐PTMC and their selective depolymerization.

Finally, the zinc complex was used in the depolymerization of the previously synthesized PTMC‐PLLA diblock copolymer (Table S3). It was observed that operating in the same conditions as entry 2 in Table 3, after 0.5 hour the PLLA was completely depolymerized to the corresponding monomer, while the PTMC block remained completely intact (entry 1 in Table S3). Following the removal of L‐LA from the reaction mixture, the depolymerization of PTMC was successfully carried out using the same catalyst. The selective depolymerization of the diblock copolymer highlights the feasibility of separately recovering both starting monomers, establishing an efficient chemical recycling process (Scheme 5). In contrast, the depolymerization of the PTMC‐PCL diblock copolymer leads to the formation of TMC, while the PCL block remains intact (entry 2 in Table S3). The same reaction carried out on a polycaprolactone homopolymer confirmed that complex **1** is unable to depolymerize PCL (entry 3 in Table S3). As a result, even in copolymers formed from these two monomers, selective recycling of the TMC monomer from the unaffected PCL block is possible.

## 3 Conclusion

This study focused on the synthesis of nontoxic metal complexes through the careful selection of both metals and ancillary ligand, aiming to promote the synthesis of sustainable and biocompatible polymers. Specifically, zinc and magnesium complexes supported by a naphthoxy‐imino pyridine ligand were synthesized, extensively characterized, and subsequently employed in the preparation of aliphatic polyesters and polycarbonates. Both complexes promoted efficient ROP of L‐LA, ε‐caprolactone and β‐butyrolactone, as cyclic esters, and TMC and 1‐methyl‐trimethylene carbonate, as cyclic carbonates.

Zinc complexes compared to magnesium complexes showed (much) higher activity in the polymerization of LLA, β‐butyrolactone, and 1‐methyl‐trimethylene carbonate, while similar activities for the two complexes were recorded with ε‐caprolactone and TMC. Based on these results, we tentatively hypothesize that steric effects play a role in slowing down the polymerization promoted by the magnesium complex, particularly with monomers that feature a methyl group in proximity to their carbonyl moiety. Moreover, the study of the kinetics of polymerization of ε‐caprolactone promoted by the magnesium complex indicates that the formation of the active species requires a certain amount of time, which is not the case for the zinc complex. Such different performances of zinc and magnesium complexes undoubtedly merit further investigation: we intend to explore the mechanistic aspects of these reactions through both experimental and computational approaches, the results of which will be published in due course.

For all the obtained polymers, molecular masses in agreement with those expected and an excellent end‐group fidelity, as found by GPC, NMR, and MALDI analysis, demonstrated the good control of the polymerization processes.

Given its exceptional activity, positioning it among the most efficient complexes in the literature for the polymerization of LLA and TMC, the zinc complex was also employed in the synthesis of both diblock and random TMC‐based copolymers with LLA and ε‐caprolactone. The formation of diblock copolymers was confirmed by DOSY spectra.

On the other hand, the characterization of the copolymers obtained through the simultaneous polymerization of the two monomers, using NMR and DSC techniques, revealed a good degree of randomness and thermal properties consistent with their composition.

Notably, the life cycle of some of the synthesized materials was closed by the same zinc complex which promoted depolymerization reactions using microwaves as a sustainable heat source. Intriguingly, the diblock copolymers underwent selective depolymerization.

## Supporting Information

Materials, characterization, procedures, NMR spectra, TGA, and DSC traces are given in supporting Information.

## Conflict of Interests

The authors declare no conflict of interest.

## Supporting information



Supporting Information

## Data Availability

The data that support the findings of this study are available from the corresponding author upon reasonable request.
