# Supplementary material for: Recyclable, Biobased Polycarbonates and Polyesters by Naphthoxy‐Imine Zinc and Magnesium Complexes
Source: Chemistry. 2025 May 27;31(35):e202501271. doi: 10.1002/chem.202501271 (PMC12188170; doi:10.1002/chem.202501271)
Supplement: Supplementary file 1 — Supporting Information [file CHEM-31-e202501271-s001.docx]

SUPPORTING INFORMATION

Recyclable, Biobased Polycarbonates and Polyesters by Naphthoxy-Imine Zinc and Magnesium Complexes

Federica Tufano,^[a]^ Maria Vittoria Galotto,^[a]^ Alfredo D’Elia,^[a]^ Federica Santulli,*^[a]^ Mina Mazzeo,^[a],[b]^ and Marina Lamberti*^[a],[b]^

[a] Dr. F. Tufano, Dr. M.V. Galotto, Dr. A. D’Elia, Dr. F. Santulli, Prof. M. Mazzeo, Prof. M. Lamberti
Department of Chemistry and Biology “Adolfo Zambelli”
University of Salerno
via Giovanni Paolo II, 132 84084 Fisciano (SA), Italy

E-mails: [fsantulli@unisa.it](mailto:fsantulli@unisa.it), [mlamberti@unisa.it](mailto:mlamberti@unisa.it)

[b] Prof. M. Mazzeo, Prof. M. Lamberti

CIRCC, Interuniversity Consortium Chemical Reactivity and Catalysis, 70126 Bari (BA), Italy

[General Information 4](#_Toc197724935)

[Synthesis and Characterization of the Ligand 6](#_Toc197724936)

[**Scheme S1:** Synthesis of the ligand LH. 6](#_Toc197724937)

[**Figure S1.** ^1^H NMR spectrum of the ligand LH (Solvent: C_6_D_6_, 400 MHz, 298 K). 6](#_Toc197724938)

[**Figure S2.** ^13^C NMR spectrum of the ligand LH (Solvent: C_6_D_6_, 150 MHz, 298 K). 7](#_Toc197724939)

[Synthesis and Characterization of the Complexes 8](#_Toc197724940)

[**Synthesis of Zinc Complex** 8](#_Toc197724941)

[**Scheme S2:** Synthesis of zinc complex **1**. 8](#_Toc197724942)

[**Figure S3.** ^1^H NMR spectrum of **1** (Solvent: C_6_D_6_, 400 MHz, 298 K). 8](#_Toc197724943)

[**Figure S4.** ^2D^ COSY spectrum of complex **1** (Solvent: C_6_D_6_, 400 MHz, 298 K). 9](#_Toc197724944)

[**Figure S5.** Enlargement of the ^2D^ NOESY spectrum of complex **1** (Solvent:C_6_D_6_, 400 MHz, 298 K). 10](#_Toc197724945)

[**Figure S6**. ^13^C NMR spectrum of complex **1** (Solvent: C_6_D_6_, 150 MHz, 298 K). 10](#_Toc197724946)

[**Synthesis of Magnesium Complex** 11](#_Toc197724947)

[**Scheme S3:** Synthesis of magnesium complex **2**. 11](#_Toc197724948)

[**Figure S7.** ^1^ H-NMR spectrum of complex **2** (Solvent: C_6_D_6_, 400 MHz, 298 K). 11](#_Toc197724949)

[**Figure S8.** Aromatic region of the ^2D^COSY spectrum of complex **2** (Solvent: C_6_D_6_, 400 MHz, 298K). 12](#_Toc197724950)

[**Figure S9.** Enlargement of the ^2D^NOESY spectrum of complex **2** (Solvent:C_6_D_6_, 400 MHz, 298 K). 12](#_Toc197724951)

[**Figure S10.** ^1^ H-NMR spectra of complex **2**with acquisition at different temperatures (Solvent: tol-d8, 600 MHz). 13](#_Toc197724952)

[**Figure S11.** ^13^C NMR spectrum of complex **2** (Solvent: C_6_D_6_, 150 MHz, 298 K). 13](#_Toc197724953)

[**Figure S12.** Aromatic region of the ^2D^ HSQC spectrum of complex **2** (Solvent: C_6_D_6_, 400 MHz, 298K). 14](#_Toc197724954)

[**Figure S13.** Aromatic region of the ^2D^ HMBC spectrum of complex **2** (Solvent: C_6_D_6_, 400 MHz, 298K). 14](#_Toc197724955)

[Ring-Opening Polymerization (ROP) of cyclic monomers. 15](#_Toc197724956)

[**Table S1**: Kinetic studies of ɛ-caprolactone polymerization with complexes **1** and **2** 15](#_Toc197724957)

[**Figure S14.** ^1^H NMR spectra of ɛ-caprolactone polymerization with complex **1**. Acquisitions every 15 min (bottom up). (Solvent: CD_2_Cl_2_, 600 MHz, 298K). 16](#_Toc197724958)

[**Scheme S4:** Ring-opening polymerization (ROP) of lactide 17](#_Toc197724959)

[**Figure S15**: ^1^H NMR spectrum of polylactide (solvent: CDCl_3_, 400 MHz, 298 K). 17](#_Toc197724960)

[**Figure S16:** MALDI-TOF mass spectrum (matrix DCTB) of the isolated polymer from L-LA polymerization. Polymerization conditions: [LLA]_0_/[^i^PrOH]_0_/[Zn] = 50:1:1 at 25 °C in CH_2_Cl_2_ solution. 17](#_Toc197724961)

[**Scheme S5:** Ring-opening polymerization (ROP) of ɛ-caprolactone 18](#_Toc197724962)

[**Figure S17:** ^1^H NMR spectrum of polycaprolactone (solvent: CDCl_3_, 400 MHz, 298 K) * = methanol. 18](#_Toc197724963)

[**Figure S18:** MALDI-TOF mass spectrum (matrix DCTB) of the isolated polymer from ɛ-CL polymerization. Polymerization conditions: [ɛ-CL]_0_/[BnOH]_0_/[Mg] = 200:1:1 at 25 °C in CH_2_Cl_2_ solution. *(entry 9, Table 1)* 18](#_Toc197724964)

[**Scheme S6:** Ring-opening polymerization (ROP) of β-butyrolactone 19](#_Toc197724965)

[**Figure S19:** ^1^H NMR spectrum of poly β-butyrolactone (solvent: CDCl_3_, 400 MHz, 298 K). 19](#_Toc197724966)

[**Figure S20:** MALDI-TOF mass spectrum (matrix DCTB) of the isolated polymer from β-BL polymerization. Polymerization conditions: [β-BL]_0_/[^i^PrOH]_0_/[Zn] = 100:1:1 at 70 °C in toluene solution. (e*ntry 6, Table 1*) 19](#_Toc197724967)

[**Scheme S7:** Ring-opening polymerization (ROP) of trimethylene carbonate 20](#_Toc197724968)

[**Table S2**: ROP of TMC with complexes **1** and **2** 20](#_Toc197724969)

[**Figure S21:** ^1^H NMR spectrum of poly trimethylene carbonate (solvent: CDCl_3_, 400 MHz, 298 K). 20](#_Toc197724970)

[Ring Opening Polymerization (ROP) of 1-methyl-trimethylene carbonate 21](#_Toc197724971)

[**Scheme S8:** Synthesis of 1-Methyl-TriMethylene Carbonate (Me-TMC) 21](#_Toc197724972)

[**Figure S22:** ^1^H NMR spectrum of the methyl-trimethylene carbonate (solvent: CDCl_3_, 400 MHz, 298 K). 21](#_Toc197724973)

[**Scheme S9:** Ring-opening polymerization (ROP) of methyl-trimethylene carbonate 22](#_Toc197724974)

[**Figure S23**: ^1^H-NMR spectrum of poly-methyl-trimethylenecarbonate (Solvent: CDCl_3_, 600 MHz, 298K). 22](#_Toc197724975)

[**Figure S24**: ^13^C NMR spectra of poly-methyl-trimethylenecarbonate synthesized using magnesium complex **2** (b) (Solvent: CDCl_3_, 100.6 MHz, 298K). (*entry 18, Table 1*) 23](#_Toc197724976)

[**Figure S25:** MALDI-TOF mass spectrum (matrix: DCTB) of the isolated polymer from methyl-trimethylene carbonate polymerization. Polymerization conditions: [Me-TMC]_0_/[BnOH]_0_/[**Mg**] = 50:1:1 at 25 °C. (*entry 18, Table 1*) 23](#_Toc197724977)

[Synthesis of Copolymers 24](#_Toc197724978)

[**Sequential copolymerization of TMC and LLA** 24](#_Toc197724979)

[**Figure S26**: ^1^H-NMR spectrum of PTMC-b-PLA (Solvent: CDCl_3_, 400 MHz, 298K). (*entry 2, Table 2*) 24](#_Toc197724980)

[**Figure S27**: DOSY spectrum of PTMC-b-PLA (Solvent: CDCl_3_, 400 MHz, 298K) 24](#_Toc197724981)

[**Figure S28**: DSC thermogram of PTMC-b-PLA sample (third run). (*entry 2, Table 2*) 25](#_Toc197724982)

[**Sequential copolymerization of ε-CL and TMC** 25](#_Toc197724983)

[**Figure S29**: ^1^H-NMR spectrum of PTMC-b-PCL (Solvent: CDCl_3_, 400 MHz, 298K). (*entry 1, Table 2*) 26](#_Toc197724984)

[**Figure S30**: DOSY spectrum of PTMC-b-PCL (Solvent: CDCl_3_, 400 MHz, 298K) 26](#_Toc197724985)

[**Figure S31**: DSC thermogram of PTMC-b-PCL sample (third run). (*entry 2, Table 2*) 26](#_Toc197724986)

[**One-pot one-step copolymerization** 27](#_Toc197724987)

[**Figure S32**: ^1^H-NMR spectrum of PTMC-co-PLA (Solvent: CDCl_3_, 400 MHz, 298K). (*entry 4, Table 2*) 27](#_Toc197724988)

[**Figure S33:** ^13^C NMR spectrum of PTMC-co-PLA (Solvent: CDCl_3_, 100.5 MHz, 298K) *(entry 4, Table 2)* 28](#_Toc197724989)

[**Figure S34**: DSC thermogram of PTMC-co-PLA sample (third run). (*entry 3, Table 2*) 28](#_Toc197724990)

[**Figure S35**: ^1^H NMR spectrum of PTMC-co-PCL (Solvent: CDCl_3_, 400 MHz, 298K). (*entry 6, Table 2)* 29](#_Toc197724991)

[**Figure S36**: ^13^C NMR spectrum of PTMC-co-PCL (Solvent: CDCl_3_, 100.5 MHz, 298K). (*entry 6, Table 2*) 29](#_Toc197724992)

[**Figure S37**: DSC thermogram of PTMC-co-PCL sample (third run). (*entry 5, Table 2*) 30](#_Toc197724993)

[Chemical recycling of PTMC using microwave irradiation 31](#_Toc197724994)

[**Figure S38:** ^1^H NMR spectrum of PTMC depolymerization with assignment of internal alkyl protons, TMC and 1,3 propanediol. (Solvent = CDCl_3_, 400 MHz, 298 K). 31](#_Toc197724995)

[**Figure S39**: Pseudo first-order kinetic plots for the consumption of PTMC by complex **1** and 1,3 propanediol. 31](#_Toc197724996)

[**Table S3**: Depolymerization of copolymers with complex **1** 32](#_Toc197724997)

# **General Information**

All the operations of synthesis and handling of air-sensitive chemicals were performed in an inert atmosphere, using Schlenk techniques and/or a glove-box in nitrogen atmosphere. The used glassware was dried in an oven at 120 °C and subsequently subjected to vacuum-nitrogen cycles. Solvents used for polymerization experiments and for the synthesis of substances instable toward air and moisture, were distilled prior to use on the opportune drying agent. In particular, THF, toluene, and benzene were dried by refluxing over sodium and benzophenone and stored under nitrogen. Dichloromethane was dried over calcium hydride and distilled prior to use. Benzyl alcohol and isopropanol were dried by refluxing over sodium. Deuterated solvents were purchased from Sigma–Aldrich and dried over activated 3-Å molecular sieves prior to use. All the reagents used for the synthesis of the complexes were purchased from Sigma Aldrich, while trimethylene carbonate was purchased from TCI. l-LA was crystallized in toluene and then dried over P_2_O_5_, ε-caprolactone and β-butyrolactone were dried over CaH_2_ and distilled under nitrogen, while TMC was purified twice by recrystallization from dry THF. The NMR spectra were recorded with BRUKER AVANCE instruments operating at 600, 400 and 300 MHz for ^1^H. Molecular masses (*M*_n_ and *M*_w_) and dispersity values (*M*_~~w~~_/*M*_n_) were measured by gel permeation chromatography (GPC), using THF as the eluent (1.0 mL min^−1^) and narrow polystyrene standards as the reference. MALDI mass spectra were recorded using a Bruker solariX XR Fourier transform ion cyclotron resonance (FT-ICR) mass spectrometer (Bruker Daltonik GmbH,Bremen, Germany) equipped with a 7 T refrigerated actively shielded superconducting magnet (Bruker Biospin, Wissembourg, France). The samples were prepared at the concentration of 1.0 mg mL^-1^ in THF, while the matrix (DCTB) was mixed at a concentration of 10.0 mg mL^-1^. Thermal analysis was carried out by using a TA-DSC Q20 apparatus manufactured by Waters/TA instruments (Waters, New Castle, DE, USA) in flowing N_2_. Polymer samples was placed into aluminum crucibles and heated/cooled runs were carried out in the range -40 - 180 °C at 10 °C/min or in the range -100 – 110 °C at 10 °C/min. All the microwave assisted reactions were performed using a microwave synthesizer from CEM Corporation (Discover 2.0).

# **Synthesis and Characterization of the Ligand**

The ligand was synthetized by condensation of the 2-hydroxy-1-naphthaldehyde (0.172 g, 1 mmol) with the 2-(2-pyridyl)ethylamine (0.122 mg, 1 mmol). The reaction was performed in reflux of ethanol (30 mL) for 4 h. The solvent was removed under vacuum, forming an orange oil. (Yield= 98%)

### **Scheme S1:** Synthesis of the ligand LH.


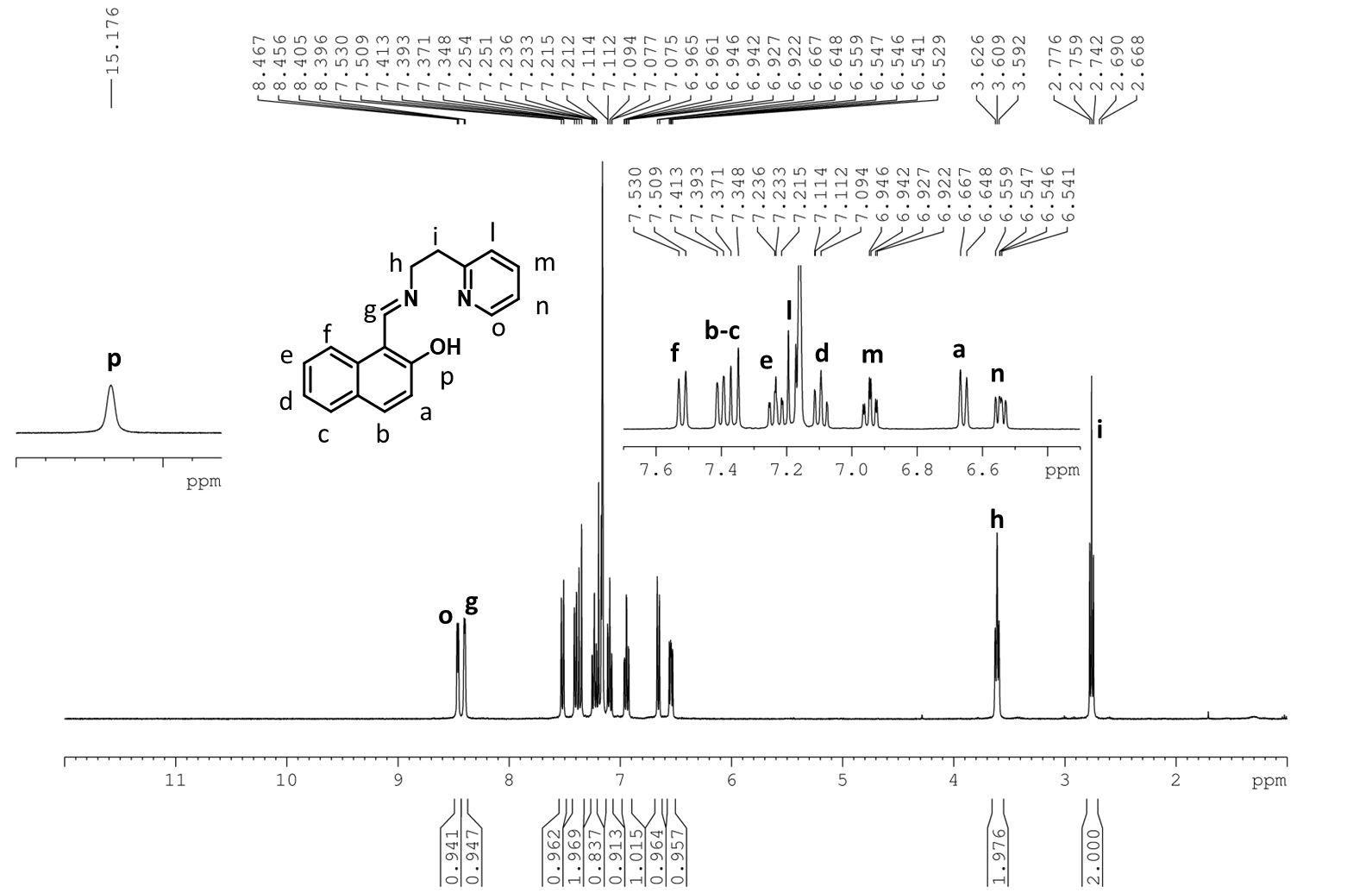


### **Figure S1.** ^1^H NMR spectrum of the ligand LH (Solvent: C_6_D_6_, 400 MHz, 298 K).

^1^H NMR (400 MHz, C_6_D_6_, 298 K): δ 15.2 (br, 1H, -OH), 8.46 (d, J = 4.5 Hz, 1H, CH-Py), 8.39 (s, 1H, CH=N), 7.51 (d, J = 8.2 Hz, 1H, CH-Ar), 7.39 (d, J = 8.0 Hz, 1H, CH-Ar), 7.34 (d, J = 9.3 Hz, 1H, CH-Ar), 7.23 (t, J = 8.0 Hz, 1H, CH-Ar), 7.17 (d, J = 9.3 Hz, 1H, CH-Ar), 7.11 (t, J = 8.0 Hz, 1H, CH-Ar), 6.93 (t, J = 7.6 Hz, 1H, CH-Ar), 6.65 (d, J = 8.2 Hz, 1H, CH-Ar), 6.55 (dd, J_1_ = 12.1 Hz, J_2_ = 5.1 Hz, 1H, CH-Ar), 3.61 (t, J = 6.7 Hz, 2H, -CH_2_), 2.74 (t, J = 6.7 Hz, 2H, -CH_2_).


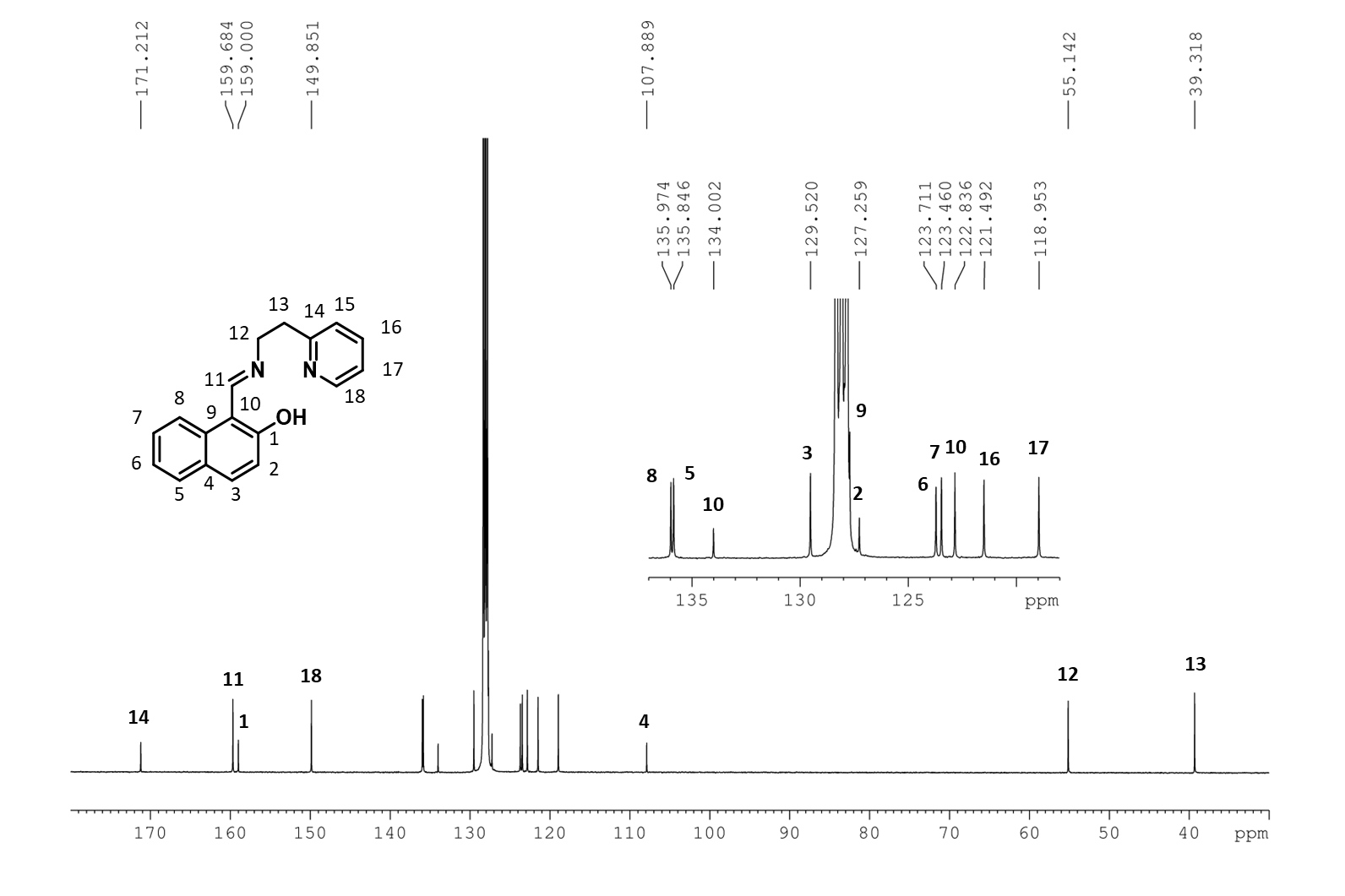


### **Figure S2.** ^13^C NMR spectrum of the ligand LH (Solvent: C_6_D_6_, 150 MHz, 298 K).

^13^C NMR (150 MHz, C_6_D_6_, 298 K): δ 171.21 (Cq), 159.68 (CH=N), 159.00 (Cq), 149.95 (Cp), 135.97 (CH), 135.84 (Cq), 134.00 (Cq), 129.52 (CH), 127.70 (Cq), 127.26 (CH), 123.71 (CH), 123.46 (CH), 122.83 (CH), 121.49 (CH), 118.95 (CH), 107.89 (Cq), 55.14 (CH_2_), 39.31 (CH_2_).

# **Synthesis and Characterization of the Complexes**

Zinc and magnesium complexes were synthesized using zinc bis [bis(trimethylsilyl) amide] Zn[N(SiMe_3_)_2_]_2_ and magnesium bis [bis(trimethylsilyl) amide] Mg[N(SiMe_3_)_2_]_2_ as metal precursors. All complexes were then characterized by NMR spectroscopy.

## **Synthesis of Zinc Complex**

### **Scheme S2:** Synthesis of zinc complex **1**.

The reaction was carried out in a glove-box in a nitrogen atmosphere. A benzene solution (8 mL) of Zn[N(SiMe_3_)_2_]_2_ (0.176 g, 4.54 * 10^-4^ mol) was added to a benzene solution (8 mL) of the ligand LH (0.200 g, 4.54 * 10^-4^ mol). Once the addition was complete, the reaction mixture was left to stir for 1h at room temperature. The solvent was then removed under reduced pressure. The obtained complex is a whitish powdery solid (Yield = 80%). Confirmation of the formation of the desired complex was obtained from the NMR spectra.


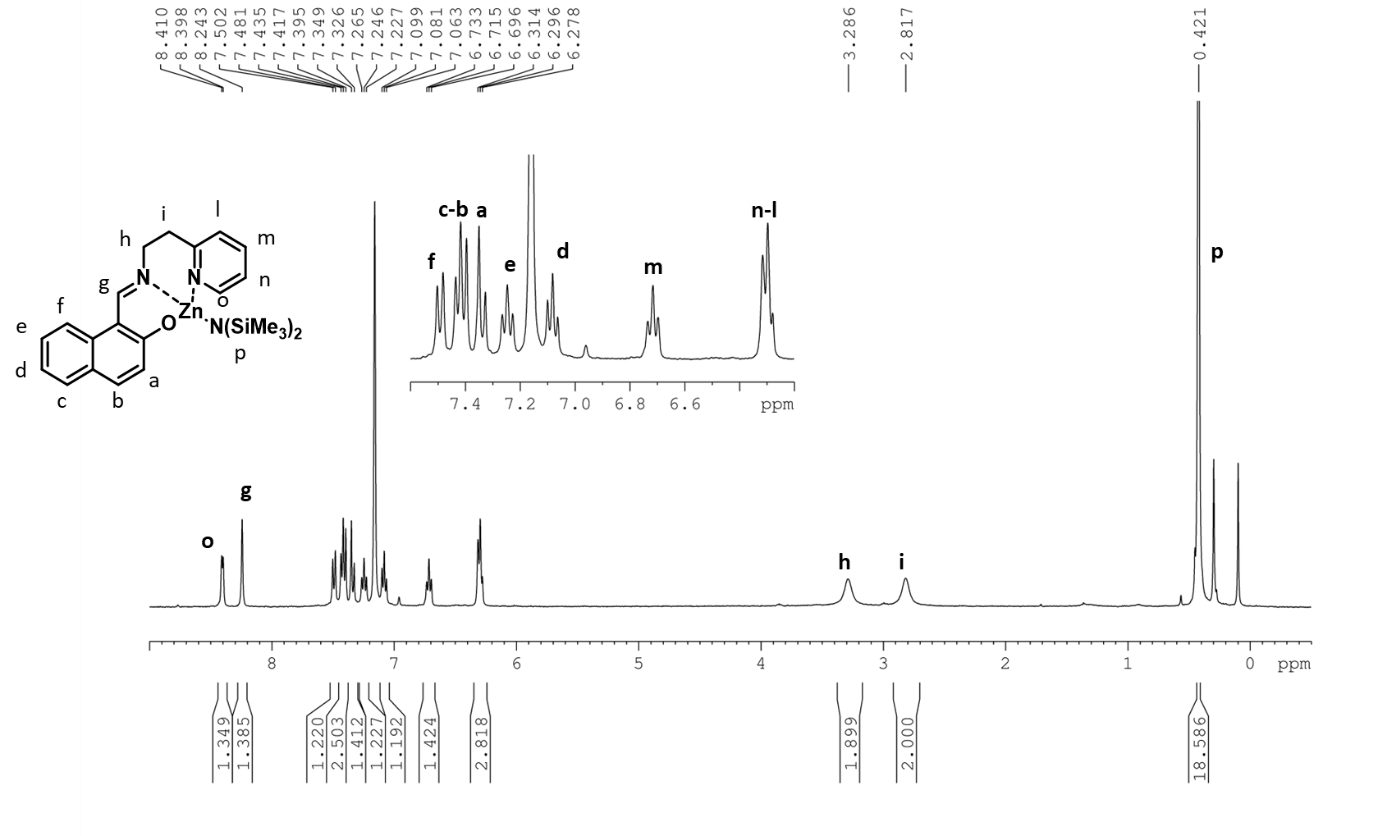


### **Figure S3.** ^1^H NMR spectrum of **1** (Solvent: C_6_D_6_, 400 MHz, 298 K).

^1^H NMR (400 MHz, C_6_D_6_, 298 K): δ 8.40 (d, J = 5.2 Hz, 1H, CH-Py), 8.24 (s, 1H, CH=N), 7.49 (d, J = 8.2 Hz, 1H, CH-Ar), 7.41 (d, J = 8.0 Hz, 1H, CH-Ar), 7.40 (d, J = 9.1 Hz, 1H, CH-Ar), 7.33 (d, J = 9.2 Hz, 1H, CH-Ar), 7.25 (t, J = 7.8 Hz, 1H, CH-Ar), 7.08 (t, J = 8.0 Hz, 1H, CH-Ar), 6.71 (t, J = 7.6 Hz, 1H, CH-Ar), 6.30 (m, 2H, CH-Ar), 3.29 (br, 2H, -CH_2_), 2.82 (br, 2H, -CH_2_), 0.42 (s, 18H, Si(CH_3_)_3_).


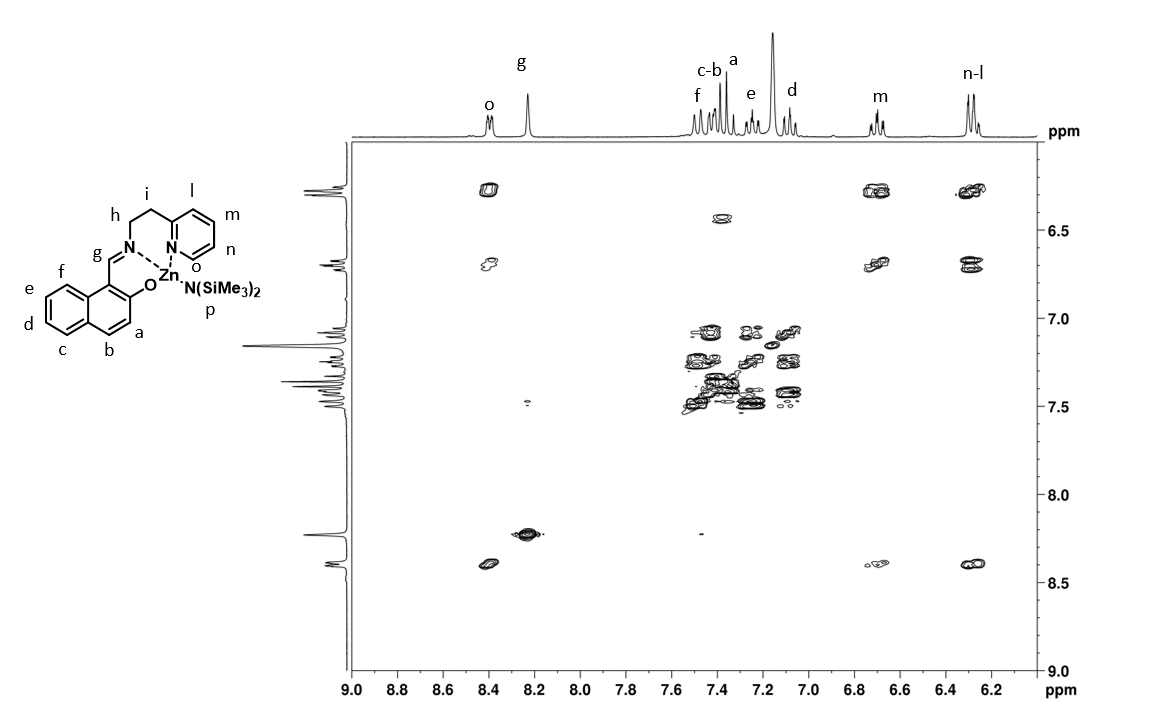


### **Figure S4.** ^2D^ COSY spectrum of complex **1** (Solvent: C_6_D_6_, 400 MHz, 298 K).


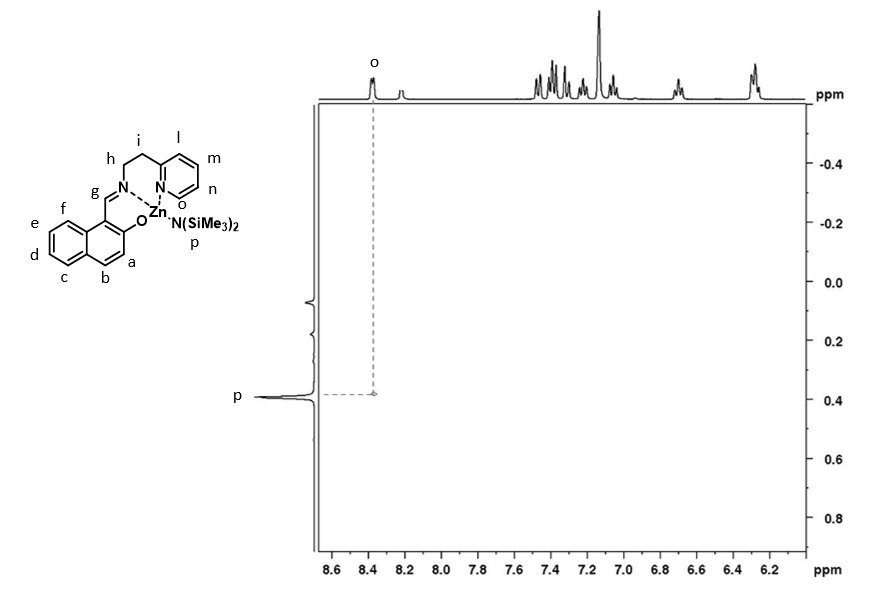


### **Figure S5.** Enlargement of the ^2D^ NOESY spectrum of complex **1** (Solvent:C_6_D_6_, 400 MHz, 298 K).


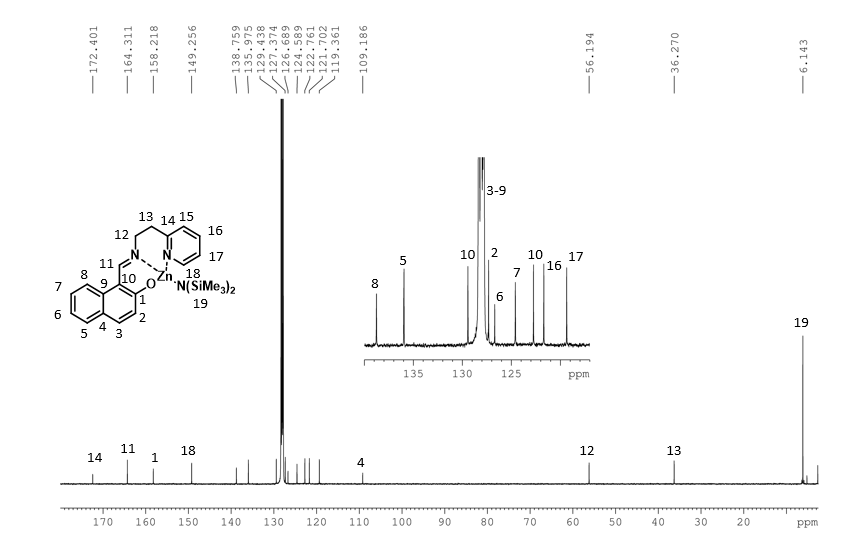


**Figure S6**. ^13^C NMR spectrum of complex **1** (Solvent: C_6_D_6_, 150 MHz, 298 K).

^13^C NMR (150 MHz, C_6_D_6_, 298 K): δ 172.40 (Cq), 164.31 (CH=N), 158.22 (Cq), 149.26 (Cp), 138.76 (CH), 135.98 (Cq), 129.44 (Cq), 128.18 (CH), 127.95 (Cq), 127.37 (CH), 126.69 (CH), 124.59 (CH), 122.76 (CH), 121.70 (CH), 119.36 (CH), 109.19 (Cq), 56.19 (CH_2_), 36.27 (CH_2_), 6.14 (Si(CH_3_)_3_).

## **Synthesis of Magnesium Complex**

### **Scheme S3:** Synthesis of magnesium complex **2**.

The reaction was carried out in a glove-box in a nitrogen atmosphere. A benzene solution (8 mL) of Mg[N(SiMe_3_)_2_]_2_ (0.157 g, 4.54 * 10^-4^ mol) was added to a benzene solution (8 mL) of the ligand LH (0.200 g, 4.54 * 10^-4^ mol). Once the addition was complete, the reaction mixture was left to stir for one hour at room temperature. The solvent was then removed under reduced pressure. The obtained complex is a whitish powdery solid (Yield = 80%). Confirmation of the formation of the desired complex was obtained from the NMR spectra.


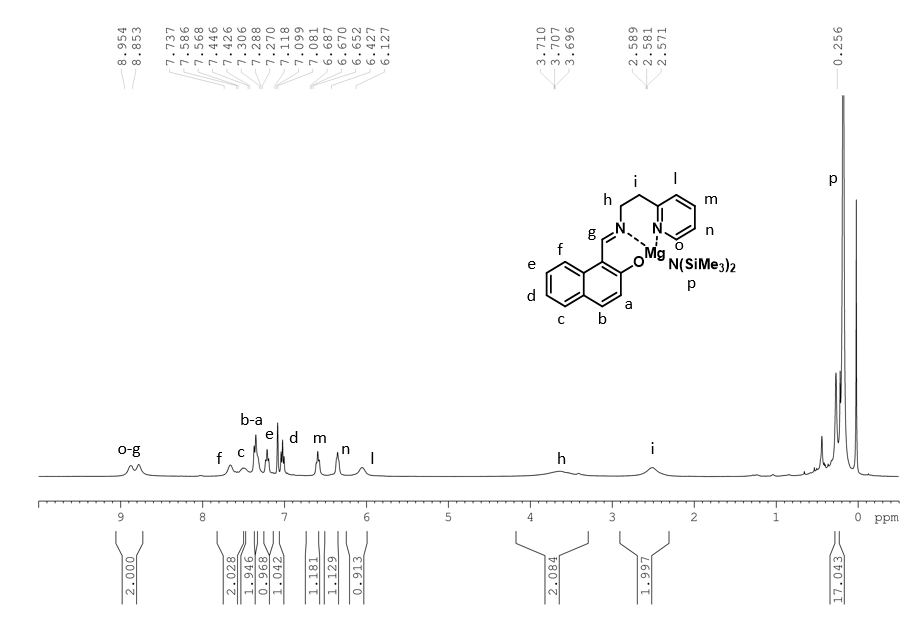


### **Figure S7.** ^1^ H-NMR spectrum of complex **2** (Solvent: C_6_D_6_, 400 MHz, 298 K).

^1^H NMR (400 MHz, C_6_D_6_, 298 K): δ 8.95-8.85 (m, 2H, CH-Ar, CH=N), 7.76 (m, 1H, CH-Ar), 7.61 (m, 1H, CH-Ar), 7.42 (m, 2H, CH-Ar), 7.30 (t, J = 7.6 Hz, 1H, CH-Ar), 7.10 (t, J = 7.8 Hz, 1H, CH-Ar), 6.67 (m, 1H, CH-Ar), 6.42 (m, 1H, CH-Ar), 6.42 (m, 1H, CH-Ar), 3.71 (br, 2H, -CH2), 2.58 (br, 2H, -CH2), 0.26 (s, 18H, Si(CH_3_)_3_).


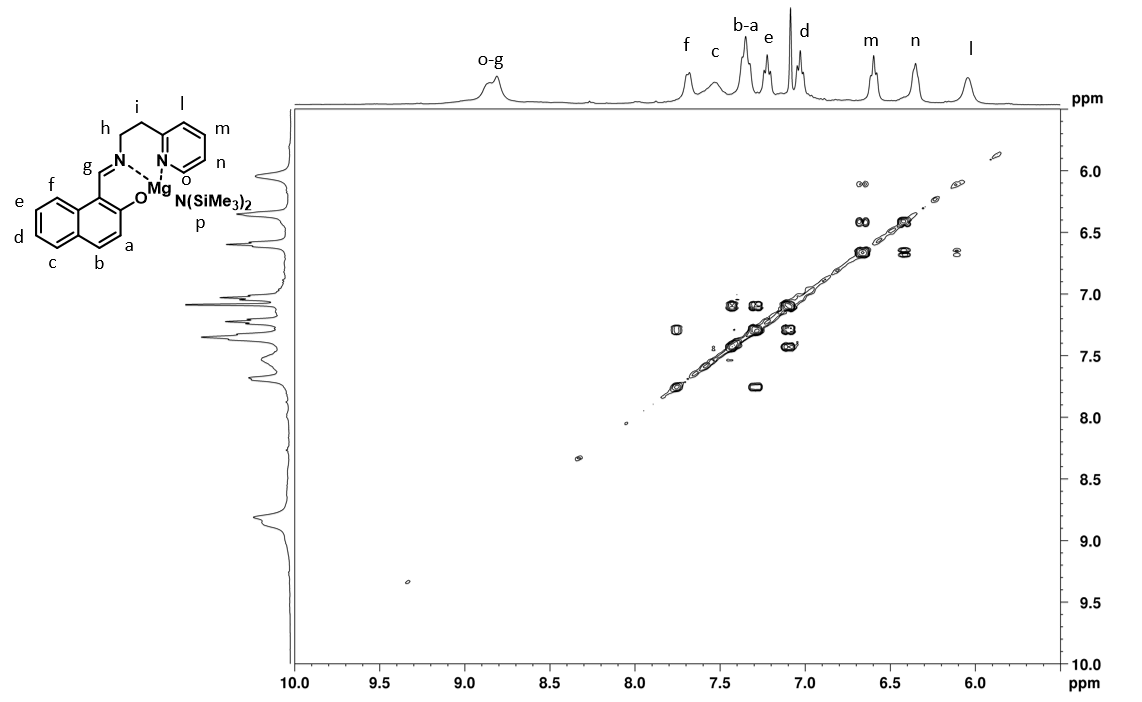


### **Figure S8.** Aromatic region of the ^2D^COSY spectrum of complex **2** (Solvent: C_6_D_6_, 400 MHz, 298K).


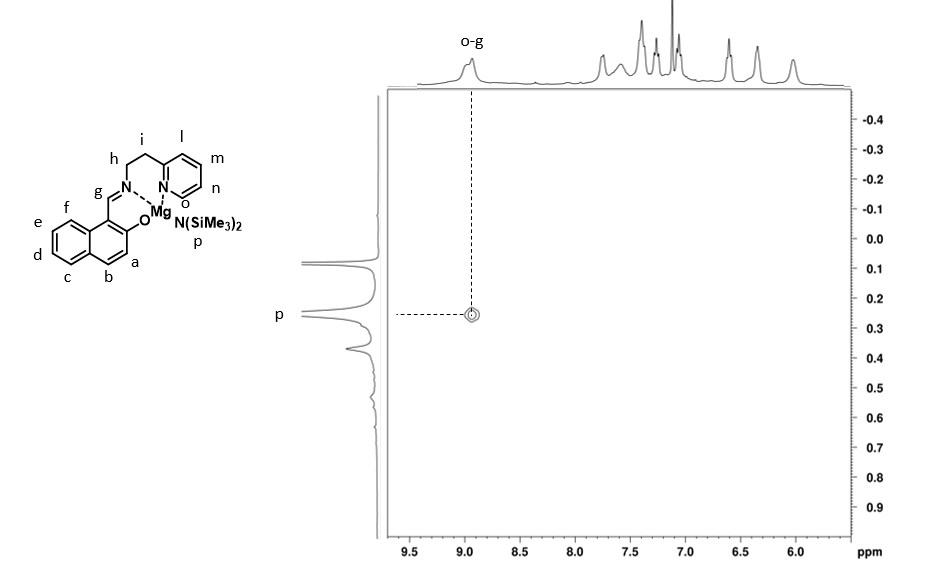


### **Figure S9.** Enlargement of the ^2D^NOESY spectrum of complex **2** (Solvent:C_6_D_6_, 400 MHz, 298 K).


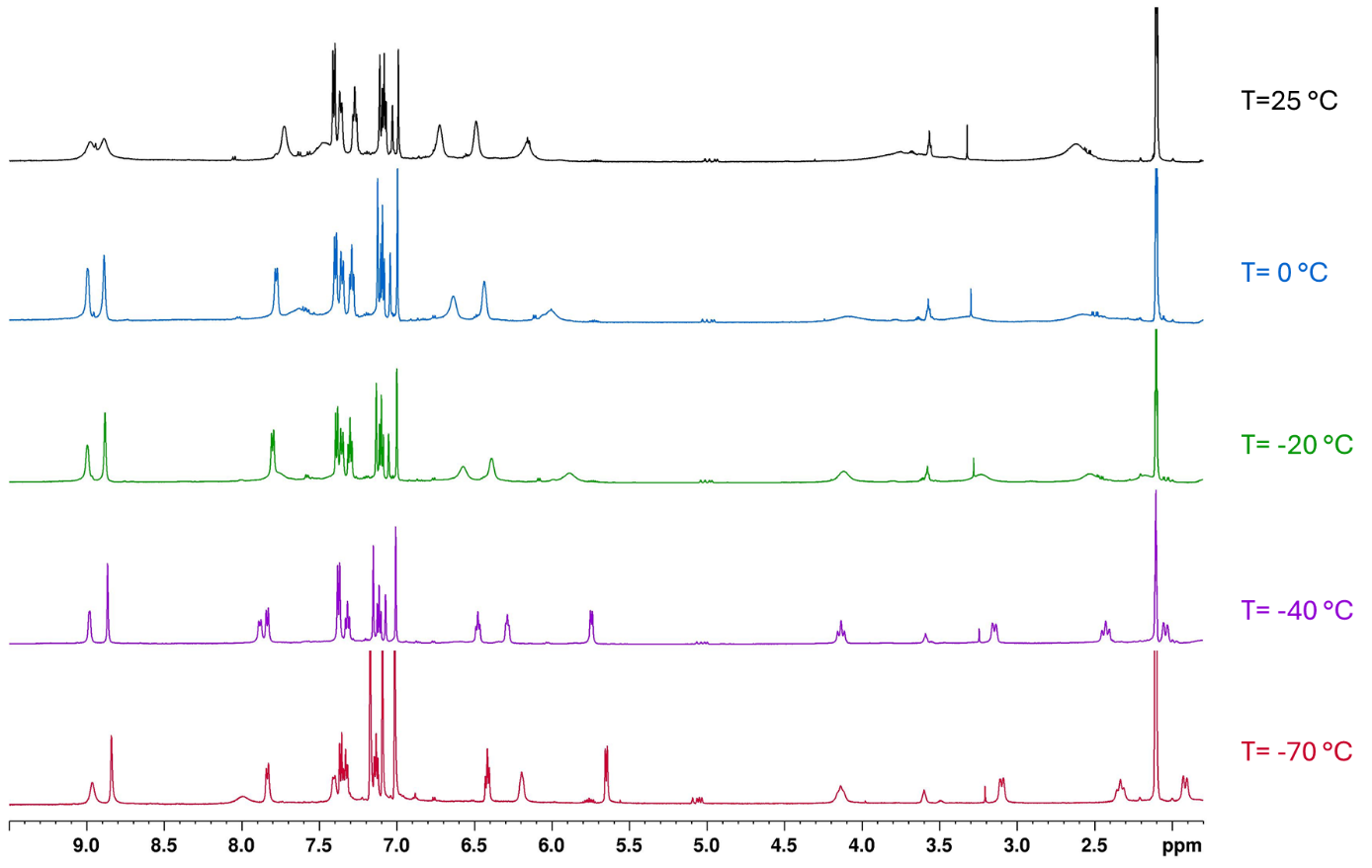


### **Figure S10.** ^1^ H-NMR spectra of complex **2**with acquisition at different temperatures (Solvent: tol-d8, 600 MHz).


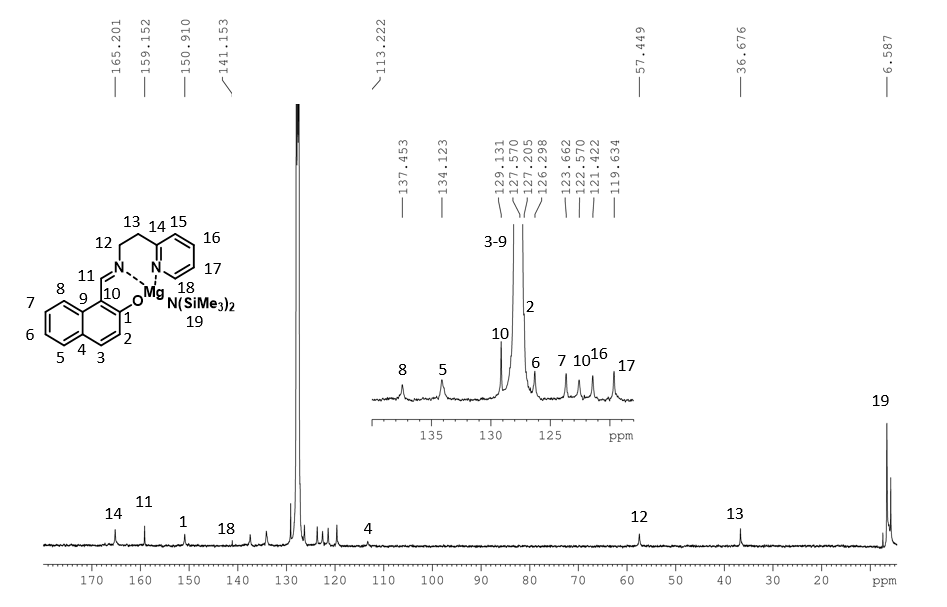


### **Figure S11.** ^13^C NMR spectrum of complex **2** (Solvent: C_6_D_6_, 150 MHz, 298 K).

^13^C NMR (150 MHz, C_6_D_6_, 298 K): δ 165.20 (Cq), 159.15 (CH=N), 150.91 (Cq), 141.15 (Cp), 137.45 (CH), 134.12 (Cq), 129.13 (Cq), 128.18 (CH), 127.57 (Cq), 127.20 (CH), 126.30 (CH), 123.66 (CH), 122.57 (CH), 121.42 (CH), 119.63 (CH), 113.22 (Cq), 57.45 (CH_2_), 36.68 (CH_2_), 6.59 (Si(CH_3_)_3_).


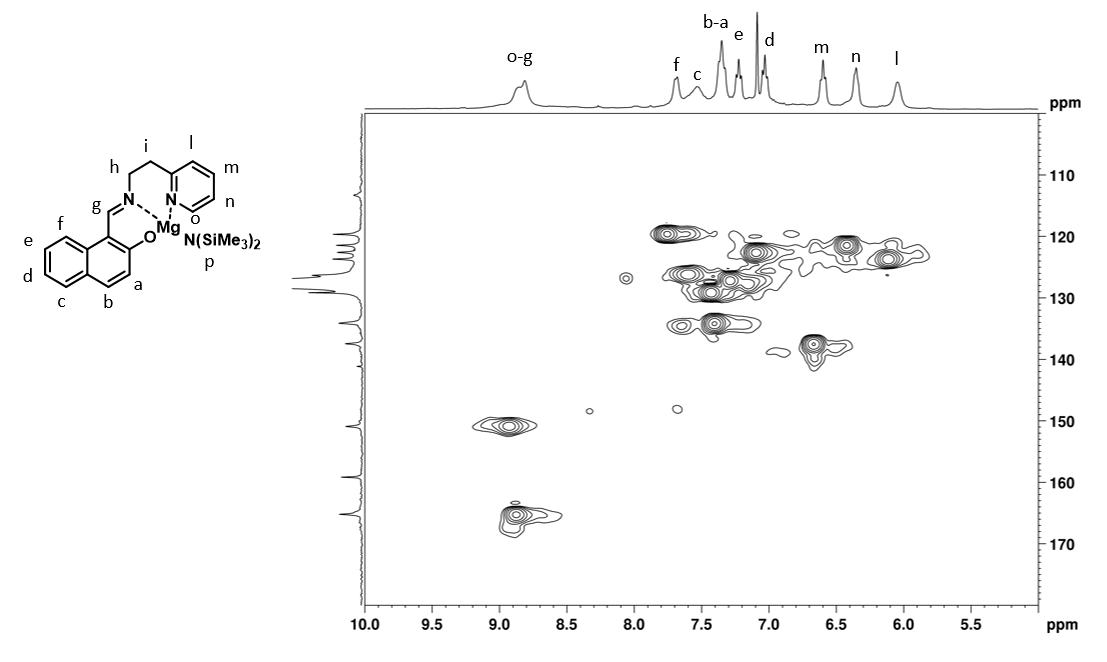


### **Figure S12.** Aromatic region of the ^2D^ HSQC spectrum of complex **2** (Solvent: C_6_D_6_, 400 MHz, 298K).


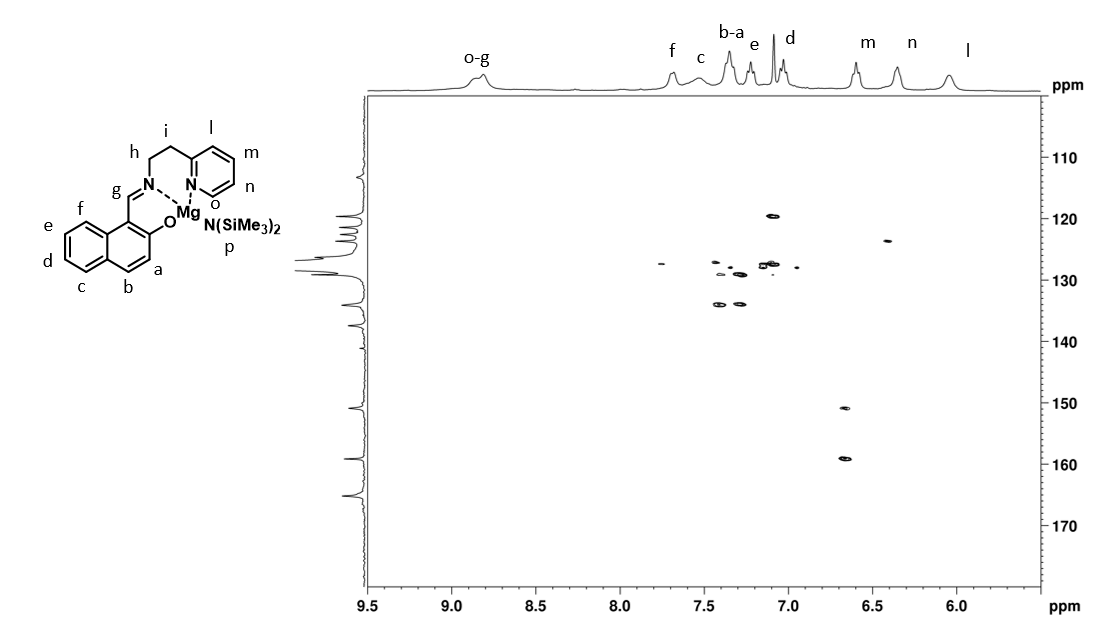


### **Figure S13.** Aromatic region of the ^2D^ HMBC spectrum of complex **2** (Solvent: C_6_D_6_, 400 MHz, 298K).

# **Ring-****Opening Polymerization (ROP) of cyclic monomers.**

**General Polymerization Procedure**

*Polymerization in solution*

The polymerization experiments were carried out in a glove-box. In a typical procedure, the complex and the monomer were weighed into two different 4 mL vials, fitted with magnetic stirrers. Both monomer and complex were dissolved in the desired solvent. Subsequently, the solution of the initiator in the same solvent used to dissolve monomer and complex, was added to the solution of the complex and left to stir for a few minutes: finally, the monomer solution was added to the reaction mixture. All the used vials were washed with solvent to recover the whole amount of each weighed compound, the total amount of solvent was 0.8 mL.

*Polymerization in bulk*

In a typical procedure, the complex was weighed into a vessel: subsequently, the initiator was added to the complex and left to stir for a few minutes, then the monomer, weighed into a 4 mL vial, was added to the reaction mixture. For the polymerization experiments conducted at higher temperature, the same procedure was followed and after the addition, the vial was closed, pulled out of the glove box and immersed in a thermostated oil bath at the desired temperature.

All the polymerization experiments were stopped using wet dichloromethane, after taking the vial out of the glovebox. The solvent was removed under reduced pressure and the polymer was washed in methanol, dried and characterized by NMR spectroscopy, MALDI mass spectrometry and/or GPC analysis.

**Kinetic studies for ɛ-caprolactone polymerization**

ɛ-caprolactone (250 µmol, 28.5 mg, 100 equiv) was weighed into a 4 mL vial, dissolved in 0.4 mL CD_2_Cl_2_, and transferred to a J-Young NMR tube. The complex (2.5 µmol, 1 equiv) was weighed into a 4 mL vial and dissolved in 0.5 mL CD_2_Cl_2_. Subsequently, 0.1 mL of iPrOH solution (0.025 M in CD_2_Cl_2_, 2.5 µmol, 1 equiv) was added to the complex solution and allowed to stir for 5 min. The reaction mixture was added to the monomer solution, and NMR acquisition was performed every 15 min.

### **Table S1**: Kinetic studies of ɛ-caprolactone polymerization with complexes **1** and **2**

| Entry | Complex | Time  (h) | Conv  (%) |
| --- | --- | --- | --- |
| 1 | **1** | 0.08  0.41  0.75  1.08  1.42  1.75  2.08  2.42  2.75  3.08 | 0.007  0.085  0.18  0.29  0.35  0.45  0.52  0.57  0.62  0.66 |
| 2 | **2** | 0.08  0.42  0.75  1.08  1.42  1.75  2.08  2.42  2.75  3.08 | 0.01  0.02  0.06  0.21  0.36  0.50  0.60  0.67  0.72  0.76 |

The reactions were performed used 2.5 µmol of complex with [ɛ-CL]: [complex]: [^i^PrOH]= 100: 1: 1 in 1.0 mL of CD_2_Cl_2_ at 25 °C.


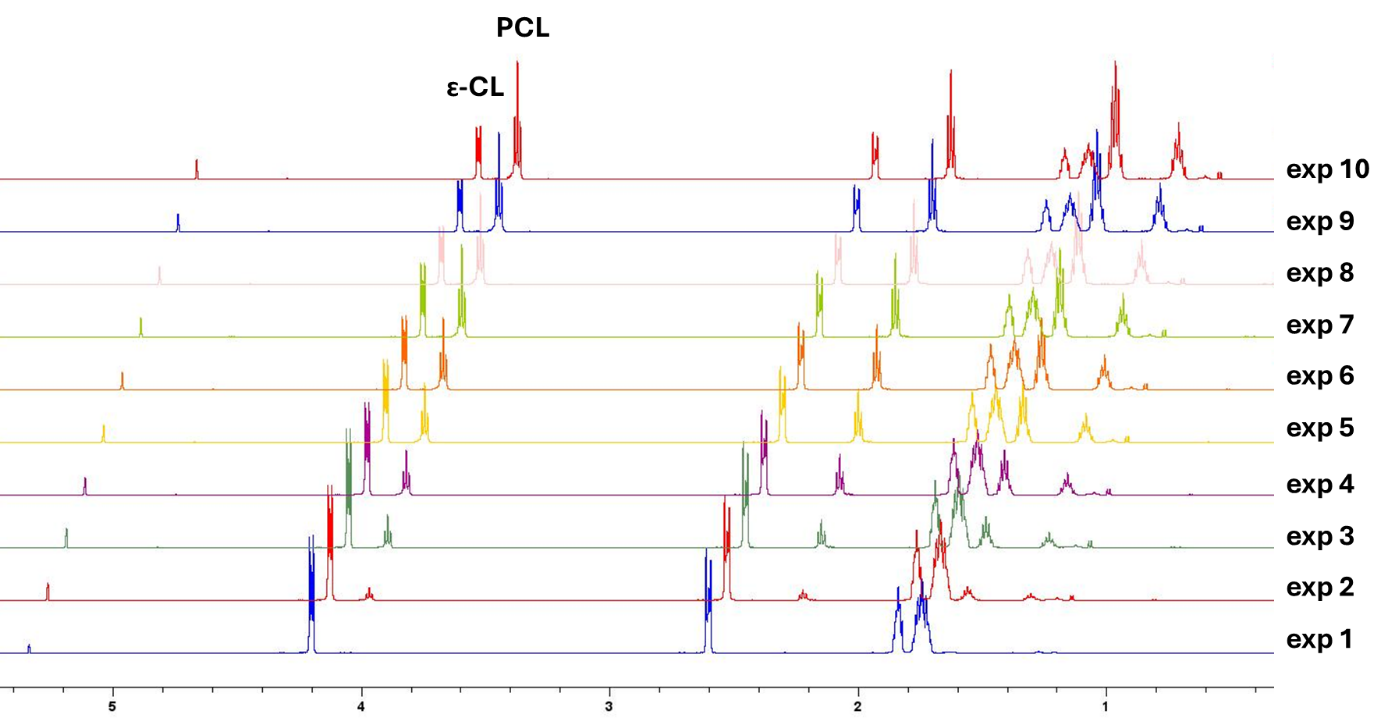


### **Figure S14.** ^1^H NMR spectra of ɛ-caprolactone polymerization with complex **1**. Acquisitions every 15 min (bottom up). (Solvent: CD_2_Cl_2_, 600 MHz, 298K).

### **Scheme S4:** Ring-opening polymerization (ROP) of lactide


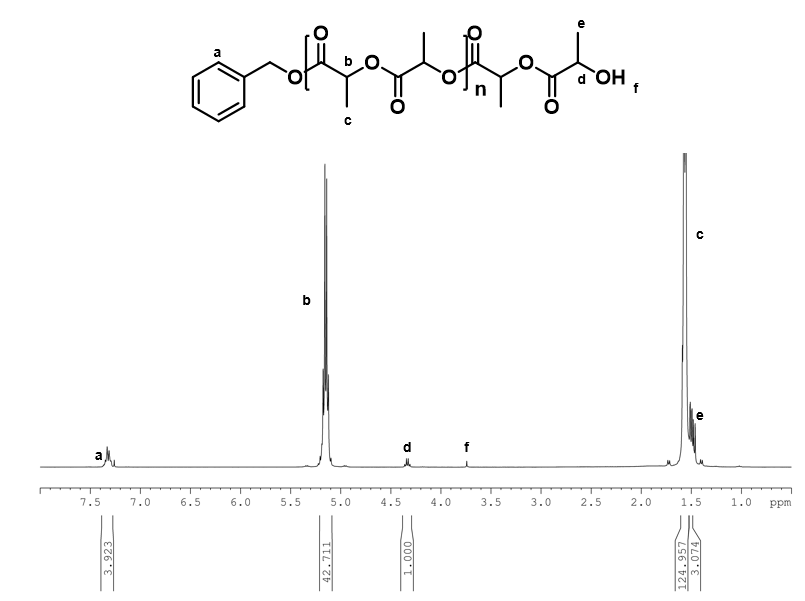


### **Figure S15**: ^1^H NMR spectrum of polylactide (solvent: CDCl_3_, 400 MHz, 298 K).


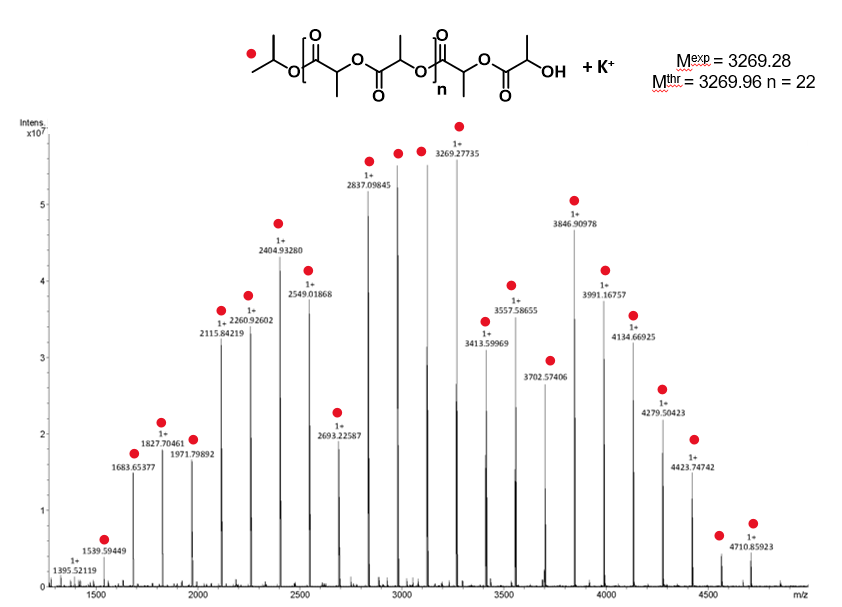


### **Figure S16:** MALDI-TOF mass spectrum (matrix DCTB) of the isolated polymer from L-LA polymerization. Polymerization conditions: [LLA]_0_/[^i^PrOH]_0_/[Zn] = 50:1:1 at 25 °C in CH_2_Cl_2_ solution.

### **Scheme S5:** Ring-opening polymerization (ROP) of ɛ-caprolactone


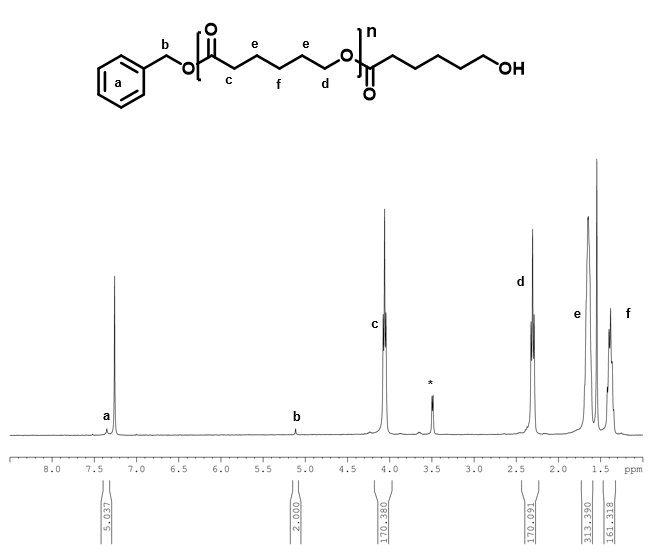


### **Figure S17:** ^1^H NMR spectrum of polycaprolactone (solvent: CDCl_3_, 400 MHz, 298 K) * = methanol.


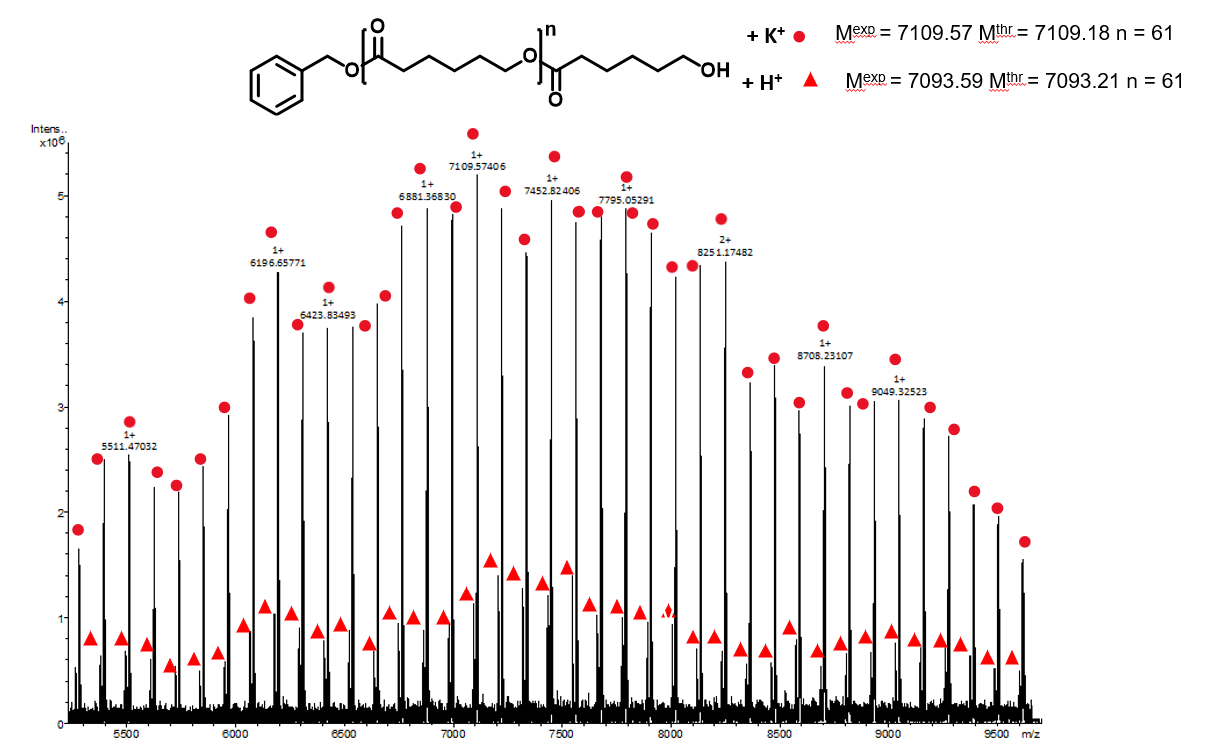


### **Figure S18:** MALDI-TOF mass spectrum (matrix DCTB) of the isolated polymer from ɛ-CL polymerization. Polymerization conditions: [ɛ-CL]_0_/[BnOH]_0_/[Mg] = 200:1:1 at 25 °C in CH_2_Cl_2_ solution. *(entry 9, Table 1)*

### **Scheme S6:** Ring-opening polymerization (ROP) of β-butyrolactone


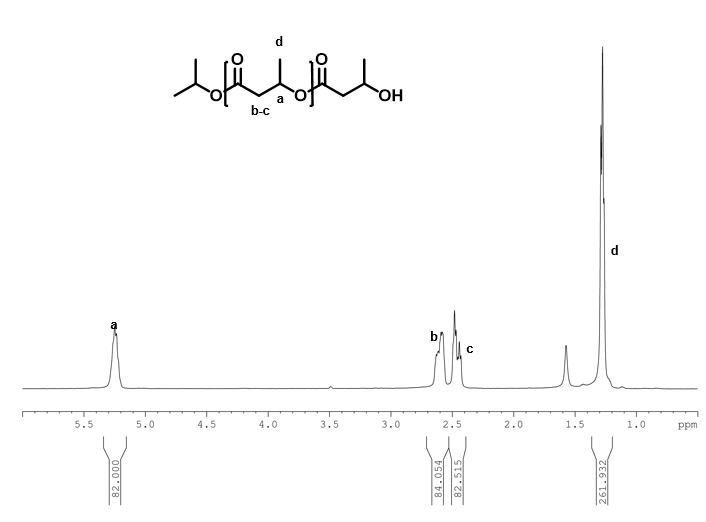


### **Figure S19:** ^1^H NMR spectrum of poly β-butyrolactone (solvent: CDCl_3_, 400 MHz, 298 K).


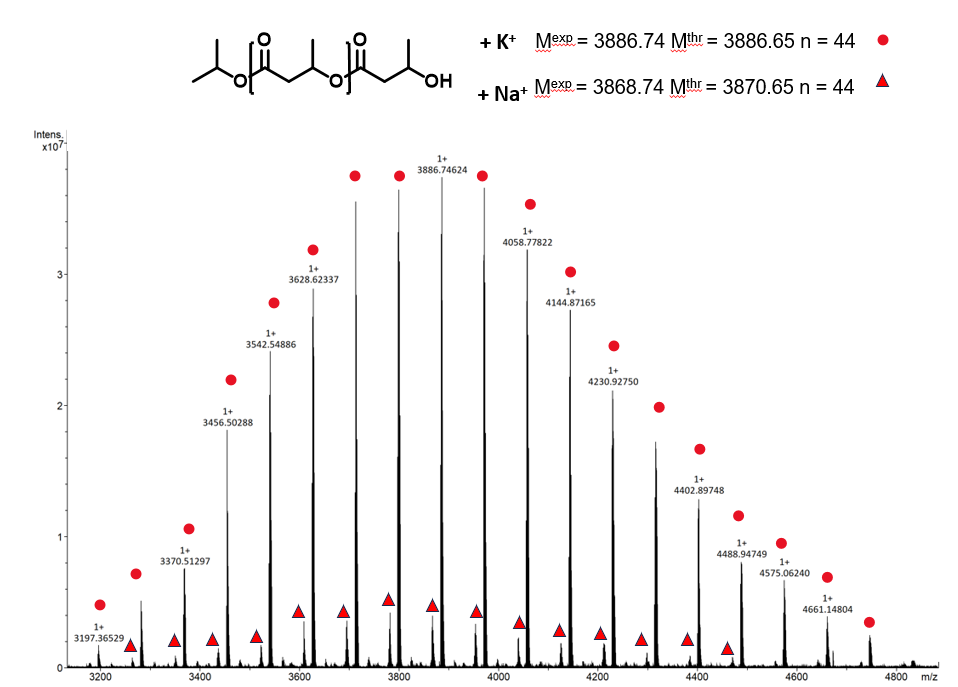


### **Figure S20:** MALDI-TOF mass spectrum (matrix DCTB) of the isolated polymer from β-BL polymerization. Polymerization conditions: [β-BL]_0_/[^i^PrOH]_0_/[Zn] = 100:1:1 at 70 °C in toluene solution. (e*ntry 6, Table 1*)

### **Scheme S7:** Ring-opening polymerization (ROP) of trimethylene carbonate

### **Table S2**: ROP of TMC with complexes **1** and **2**

| Entry^[a]^ | Complex  (μmol) | TMC  (eq)  [mol/L] | Time | Conv  (%) | *M*_n_^th [b]^ (kDa) | *M*_n_^GPC *[c]^ (kDa) | Ð | Solvent  (mL) | TOF  (h^-1^) |
| --- | --- | --- | --- | --- | --- | --- | --- | --- | --- |
| 1 | **2**  8 μmol | 100  [1] | 1’  5’ | 82  88 | 8.4  9.0 | 8.8 | 1.6 | CH_2_Cl_2_  (0.8) | 4920 |
| 2 | **2**  4 μmol | 200  [1] | 1’  7’  15’ | 69  86  89 | 18.1 | 16.7 | 1.5 | CH_2_Cl_2_  (0.8) | 8280 |
| 3 | **2**  4 μmol | 200  [2] | 1’  5’ | 82  95 | 19.4 | 20.3 | 1.7 | CH_2_Cl_2_  (0.4) | 9840  2289 |
| 4 | **1**  4 μmol | 200  [2] | 5’ | 89 | 18.1 | 16.3 | 1.6 | CH_2_Cl_2_  (0.4) | 2145 |
| 5 | **2**  4 μmol | 200  [2] | 30’  1h 30’ | 73  82 | 16.7 | 16.5 | 1.6 | Dioxolane  (0.4) | 292 |
| 6 | **1**  4 μmol | 200  [2] | 30’  1h 30’ | 52  87 | 17.8 | 11.8 | 2.0 | Dioxolane  (0.4) | 208 |

^[a]^ General conditions: initiator BnOH= 1 equivalent, (premixing cat/init for 5 min); ^[b]^ *M*_n_^th^= 102.09 · ([TMC]/[BnOH]) · conversion TMC; ^[c]^*M*_n_^GPC^*= *M*_n_^GPC^ corrected by a factor of 0.73 for 5 <*M*_n_<10 kDa, 0.88 for *M*_n_>10 kDa; ^[d]^TOF= eq x conv/time


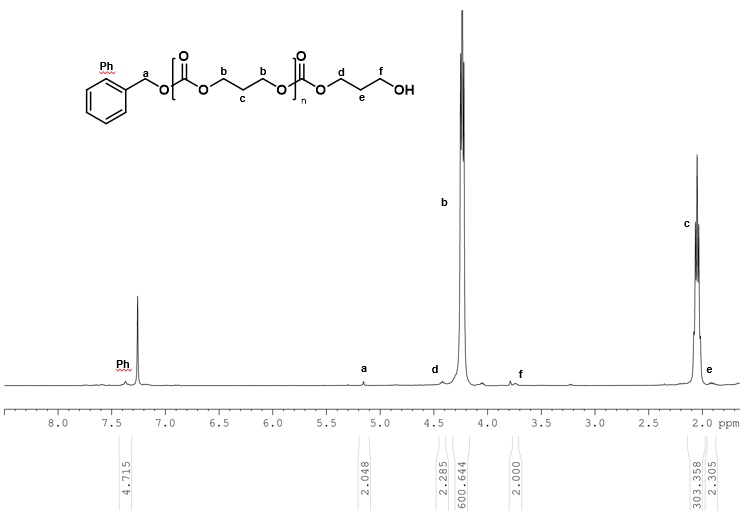


### **Figure S21:** ^1^H NMR spectrum of poly trimethylene carbonate (solvent: CDCl_3_, 400 MHz, 298 K).

# **Ring Opening Polymerization (ROP) of 1-methyl-trimethylene carbonate**

Following literature procedures, 1-methyl-trimethylene carbonate (1-Me-TMC) was prepared from CO_2_ and 1-3 butanediol.

### **Scheme S8:** Synthesis of 1-Methyl-TriMethylene Carbonate (Me-TMC)

In a flask, a 0.40 mol L^–1^ solution of 1,3-butanediol (1.70 mmol) and tosyl chloride (TsCl, 325 mg, 1.70 mmol, 1 equiv.) in anhydrous acetonitrile (4.3 mL) is prepared. The atmosphere of the flask is exchanged for CO_2_ and the solution saturated with CO_2_. Under a continuous feed of gas, 2,2,6,6-tetramethylpiperidine (TMP, 575 μL, 3.40 mmol, 2 equiv.) is added dropwise at 0 °C, then the reaction is left to reach room temperature under stirring. After approximately 20 minutes, a white precipitate forms and CO_2_ stopped being fed to the vessel. After 20 hours, the reaction mixture is diluted with non-anhydrous acetonitrile (10 mL) and the liquid phase is separated by centrifugation (3 x 5 minutes at 3000 rpm). The solvent is then removed *in vacuo.* Purification by column chromatography (diameter of column = 1.5 cm, product: silica = 1: 19, 1:1 EtOAc:Hex) affords the cyclic carbonate product (Yield % _Me-TMC_ = 70%).


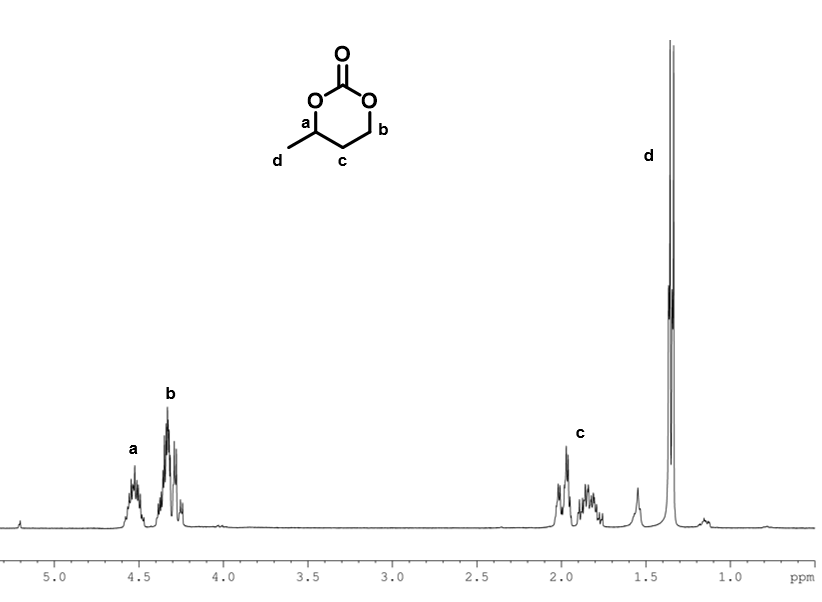


### **Figure S22:** ^1^H NMR spectrum of the methyl-trimethylene carbonate (solvent: CDCl_3_, 400 MHz, 298 K).

^1^H NMR (400 MHz, CDCl_3_, 298 K): δ 4.68-4.44 (m, 1H, OCH), 4.46-4.21 (m, 2H, OCH_2_), 2.06-1.88 (m, 2H, CH_2_), 1.35 (d, 3H, CH_3_).

The polymerization experiments in bulk were carried out in a glove box or in a thermostated bath, at desired temperature. In a typical procedure, the complex (4 x 10^-6^ mol) was weighed into a vessel. Subsequently, the initiator was added to the complex and left to stir for a few minutes and the monomer, weighed into a 4 mL vial, was added to the reaction mixture. Conversions have been determined by ^1^H NMR spectroscopy, with the quantities of polymers and monomers determined by integrating the proper resonances (MeTMC: δ = 4.65 ppm; P(MeTMC): δ = 4.90 ppm). All the polymerization experiments were stopped using wet dichloromethane, after taking the vial out of the glovebox. The solvent was removed under reduced pressure and the polymer was washed with methanol, dried and characterized by NMR spectroscopy, MALDI mass spectrometry and/or GPC analysis.


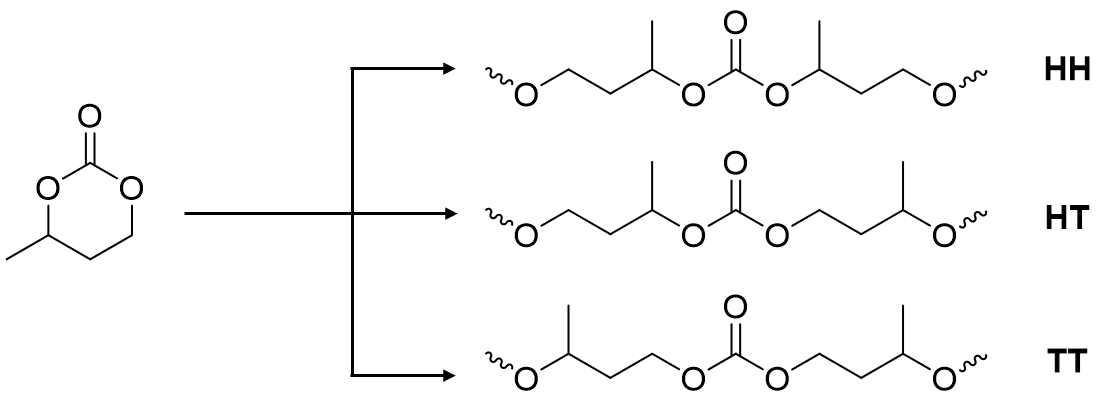


### **Scheme S9:** Ring-opening polymerization (ROP) of methyl-trimethylene carbonate

**
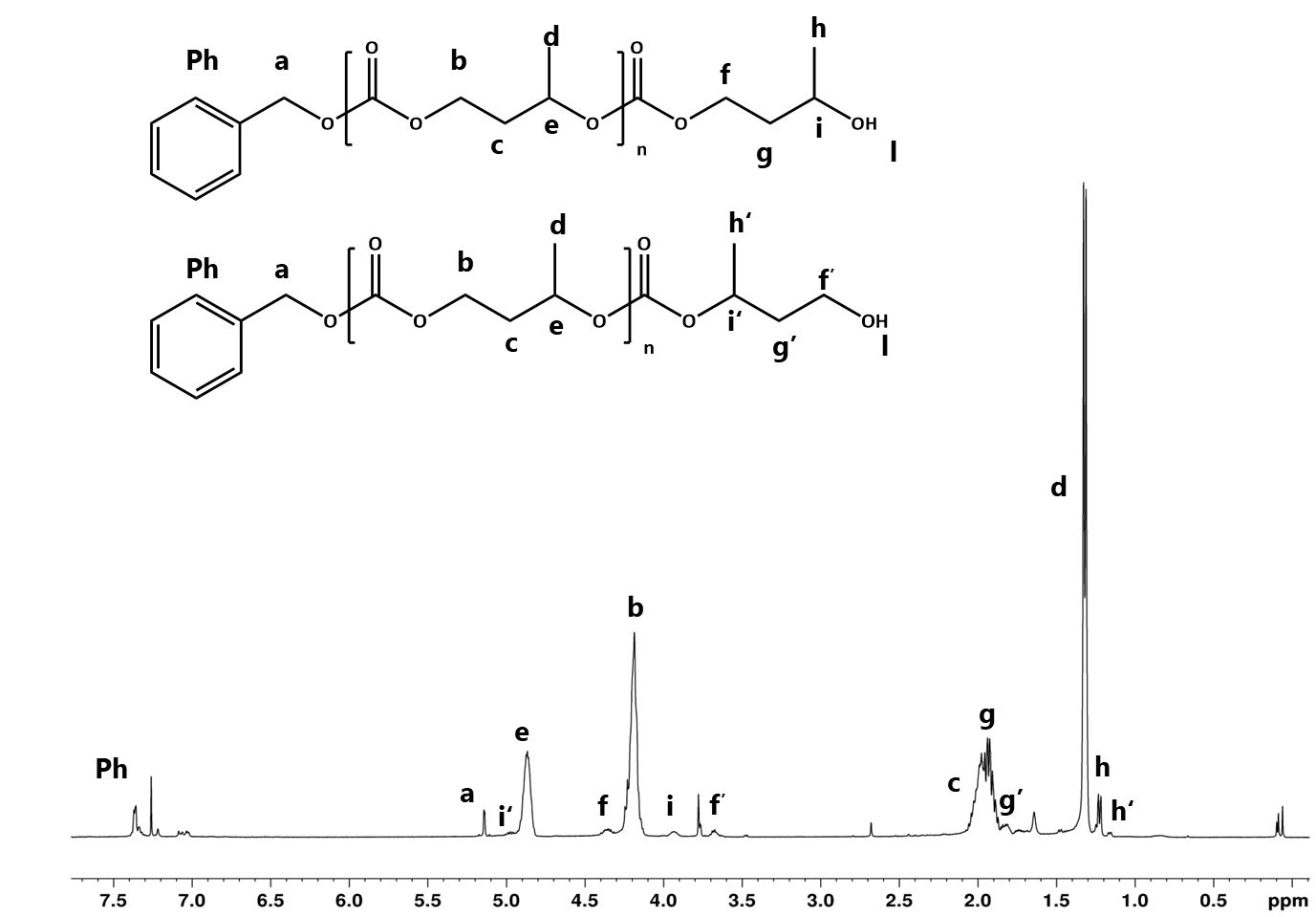
**

### **Figure S23**: ^1^H-NMR spectrum of poly-methyl-trimethylenecarbonate (Solvent: CDCl_3_, 600 MHz, 298K).


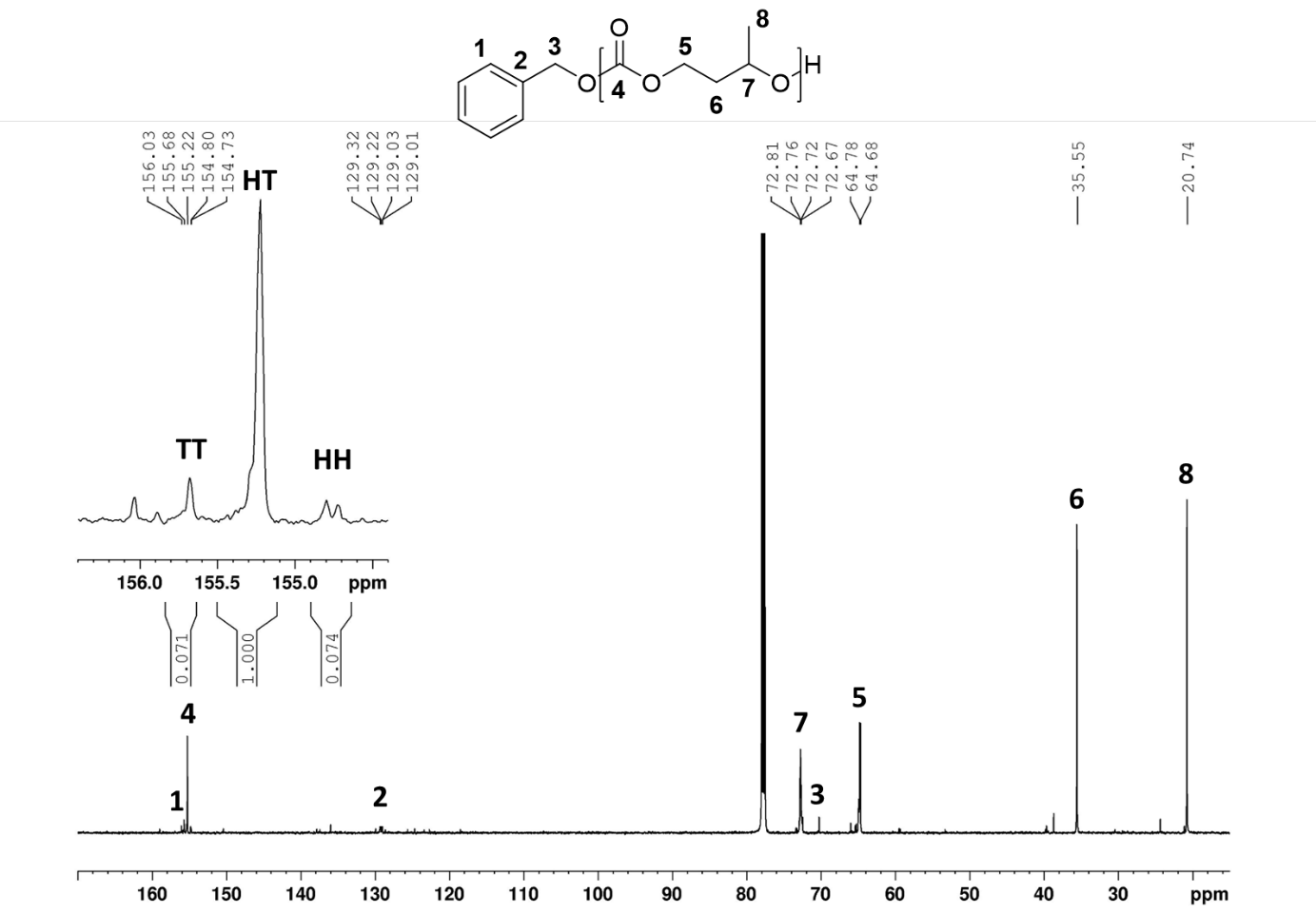


### **Figure S24**: ^13^C NMR spectra of poly-methyl-trimethylenecarbonate synthesized using magnesium complex **2** (b) (Solvent: CDCl_3_, 100.6 MHz, 298K). (*entry 18, Table 1*)


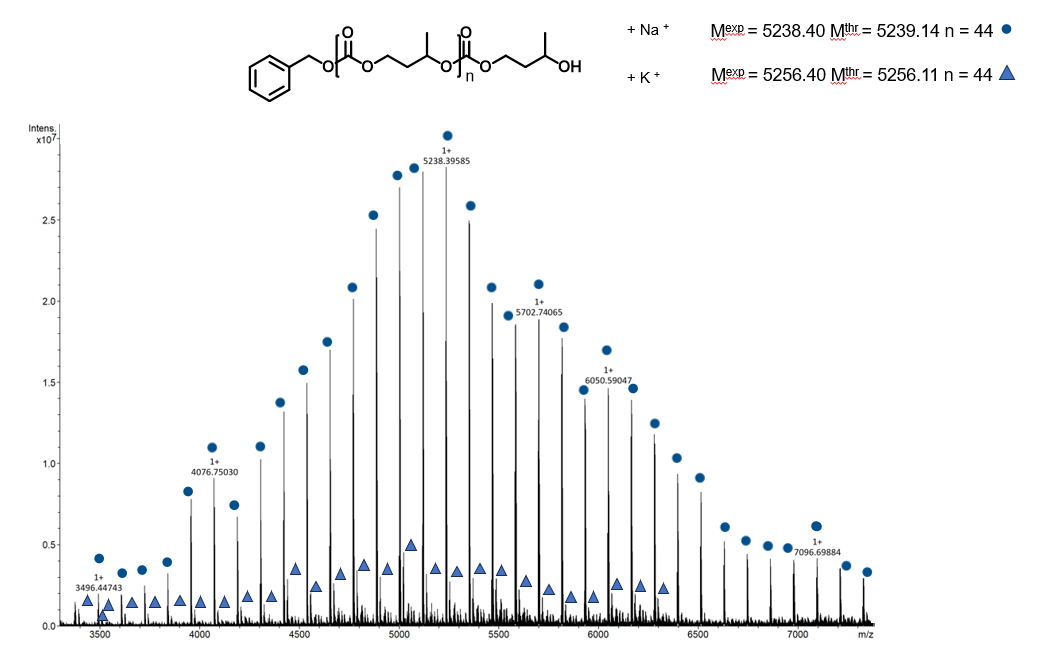


### **Figure S25:** MALDI-TOF mass spectrum (matrix: DCTB) of the isolated polymer from methyl-trimethylene carbonate polymerization. Polymerization conditions: [Me-TMC]_0_/[BnOH]_0_/[**Mg**] = 50:1:1 at 25 °C. (*entry 18, Table 1*)

# **Synthesis of Copolymers**

## **Sequential copolymerization of TMC and LLA**

Complex **1** (4.0 x 10^-6^ mol) and both monomers were weighed into three different 4 mL vials, fitted with magnetic stirrers. Monomers and complex were dissolved in DCM (0.8 mL total). 0.08 mL of a solution of BnOH (0.05 M in DCM) were added to the solution of the complex and left to stir for ten minutes. Subsequently, the trimethylene carbonate solution was introduced into the reaction mixture and allowed to stir for 5 minutes, during which the polymerization of TMC took place. Then, the solution of lactide was added to the reaction mixture. After further 5 min necessary for the LA polymerization, the sequential copolymerization was stopped using wet dichloromethane. The solvent was removed under reduced pressure and the polymer was washed in methanol, dried and characterized by NMR spectroscopy, MALDI mass spectrometry and/or GPC analysis.


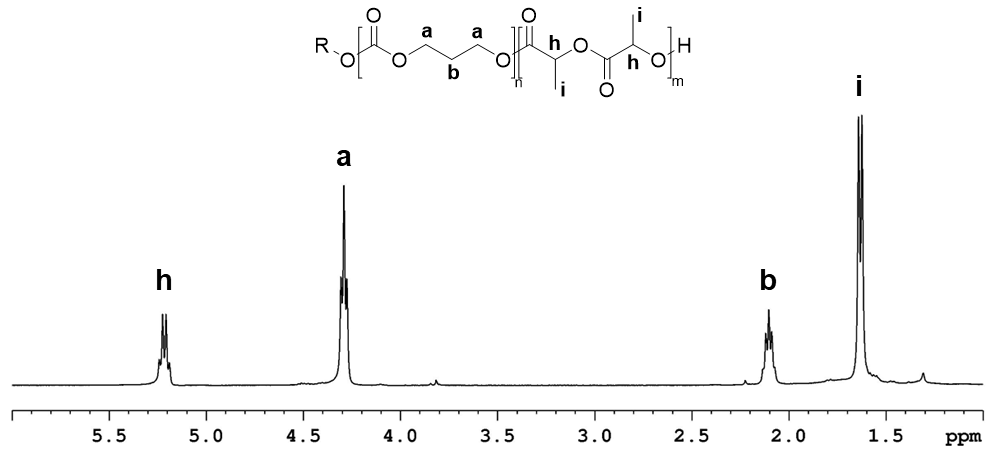


### **Figure S26**: ^1^H-NMR spectrum of PTMC-b-PLA (Solvent: CDCl_3_, 400 MHz, 298K). (*entry 2, Table 2*)


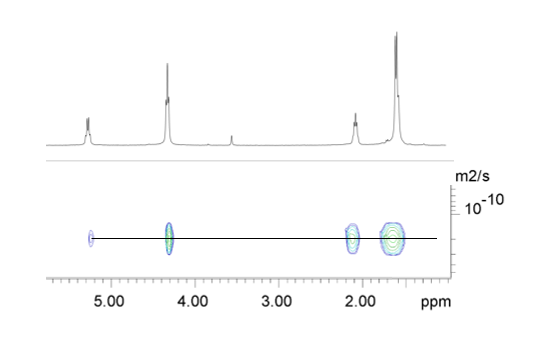


### **Figure S27**: DOSY spectrum of PTMC-b-PLA (Solvent: CDCl_3_, 400 MHz, 298K)


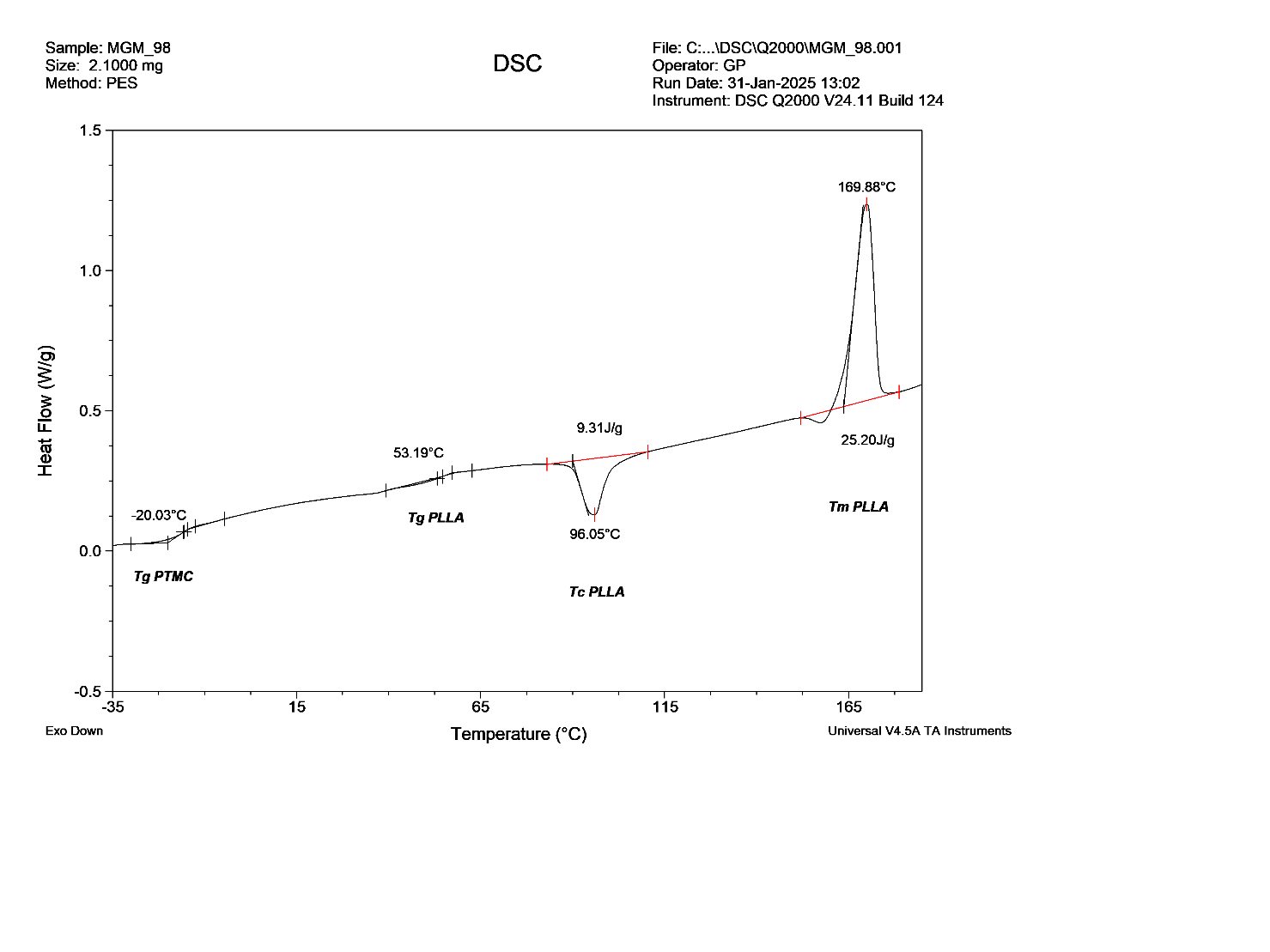


### **Figure S28**: DSC thermogram of PTMC-b-PLA sample (third run). (*entry 2, Table 2*)

## **Sequential copolymerization of ε-CL and TMC**

Complex **1** (4.0 x 10^-6^ mol) and both monomers were weighed into three different 4 mL-vials, fitted with magnetic stirrers. Monomers and complex were dissolved in DCM (0.8 mL total). 0.08 mL of a solution of BnOH (0.05 M in DCM) were added to the solution of the complex and left to stir for ten minutes. Subsequently, the solution of trimethylene carbonate was added to the reaction mixture and left to stir. When the TMC was supposed to be fully consumed (5 min), the solution of ε-caprolactone was added to the reaction mixture. After 2 h necessary for the ε-CL polymerization, the sequential copolymerization was stopped using wet dichloromethane. The solvent was removed under reduced pressure and the polymer was washed in methanol, dried and characterized by NMR spectroscopy, MALDI mass spectrometry and/or GPC analysis.


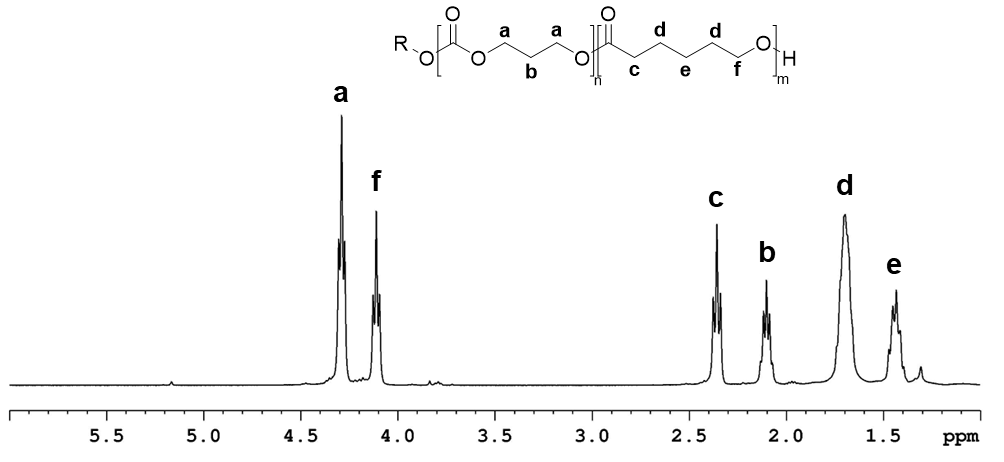


### **Figure S29**: ^1^H-NMR spectrum of PTMC-b-PCL (Solvent: CDCl_3_, 400 MHz, 298K). (*entry 1, Table 2*)


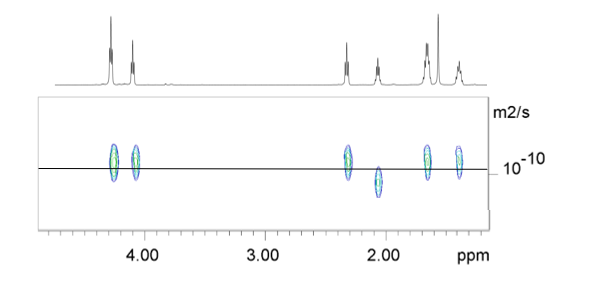


### **Figure S30**: DOSY spectrum of PTMC-b-PCL (Solvent: CDCl_3_, 400 MHz, 298K)


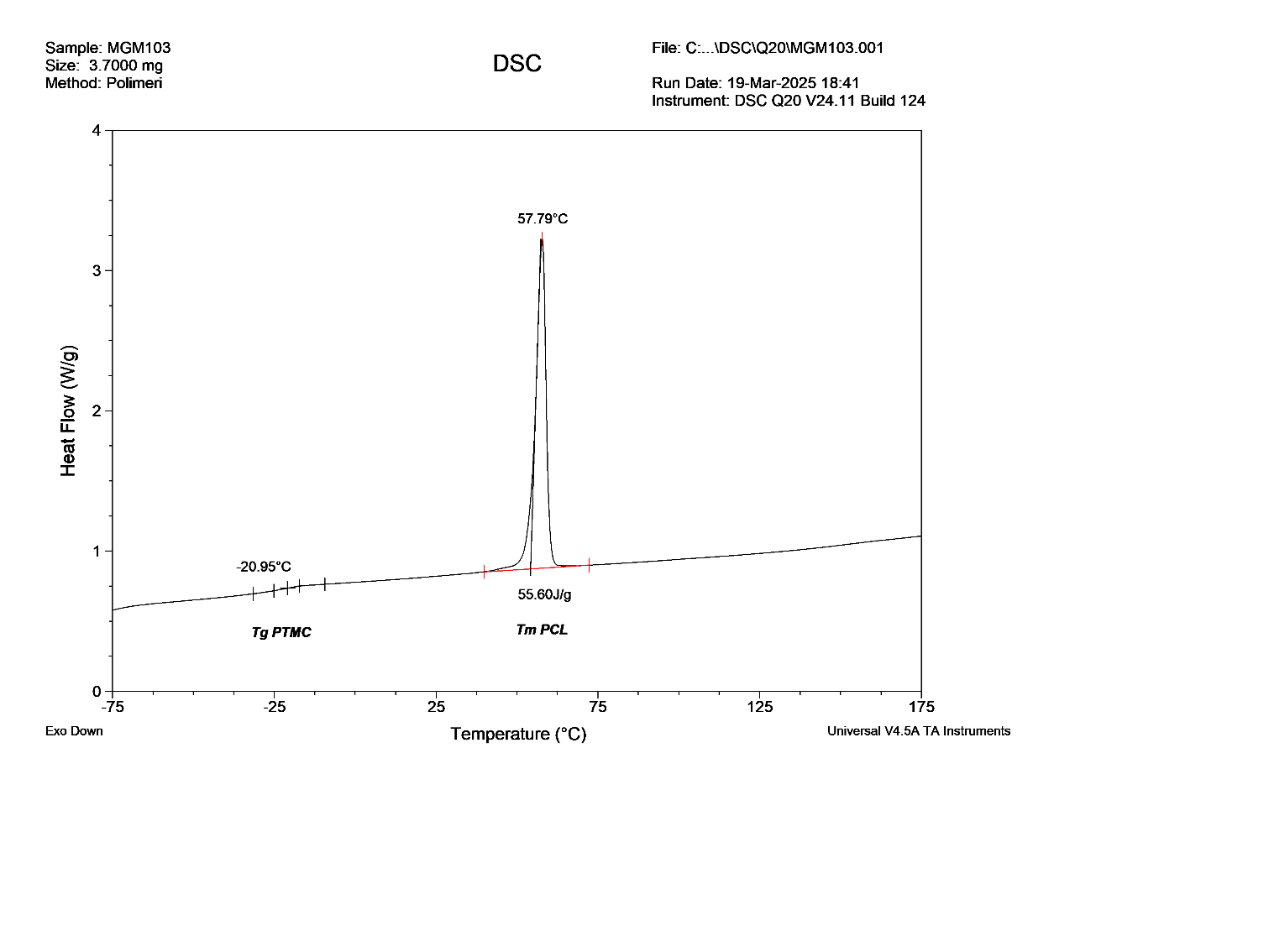


### **Figure S31**: DSC thermogram of PTMC-b-PCL sample (third run). (*entry 2, Table 2*)

## **One-pot one-step copolymerization**

In a typical procedure, complex **1** was weighed into a 4 mL vial (2 mg, 4.0 x 10^-6^ mol), and one equivalent of benzyl alcohol (stock solution in THF, 0.0185 M) was added to the complex and left to stir for few minutes. Subsequently, TMC and the other monomer (ɛ-CL or L-LA) were weighed into a 4 mL vial and placed into a thermostated oil bath at 110°C. After melting the contents of the vial, the solution of the complex and the initiator was added to the reaction mixture and left to stir, until the end of the reaction and finally cooled rapidly at 0°C. The copolymers thus obtained were purified by dissolution in minimal amount of chloroform and precipitation into cold methanol, dried and characterized by NMR spectroscopy, GPC and DSC analysis.


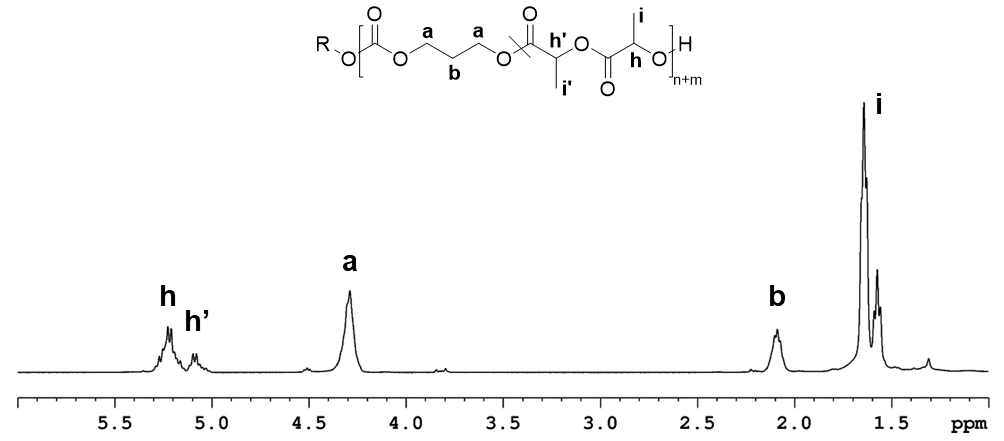


### **Figure S32**: ^1^H-NMR spectrum of PTMC-co-PLA (Solvent: CDCl_3_, 400 MHz, 298K). (*entry 4, Table 2*)


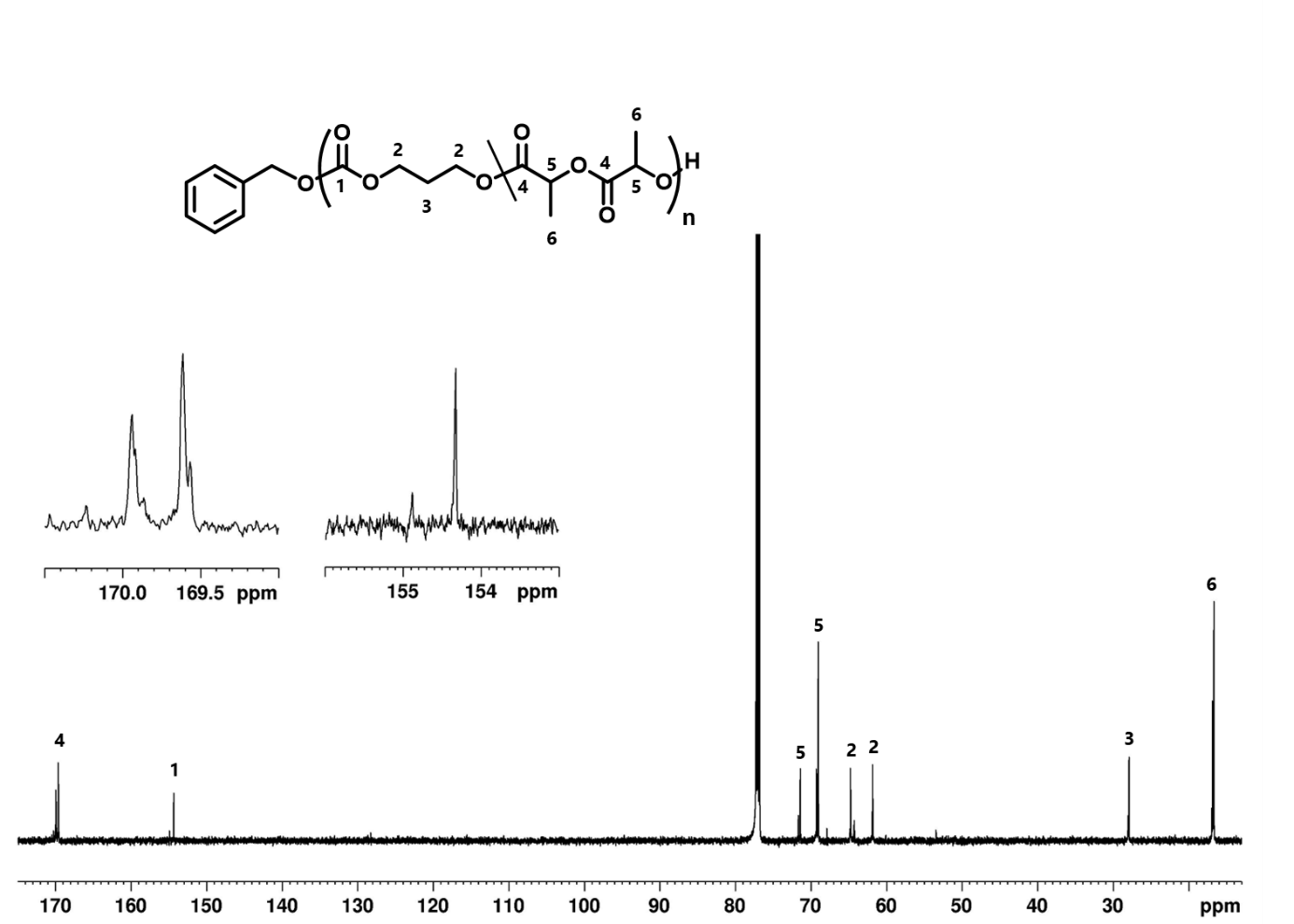


### **Figure S33:** ^13^C NMR spectrum of PTMC-co-PLA (Solvent: CDCl_3_, 100.5 MHz, 298K) *(entry 4, Table 2)*


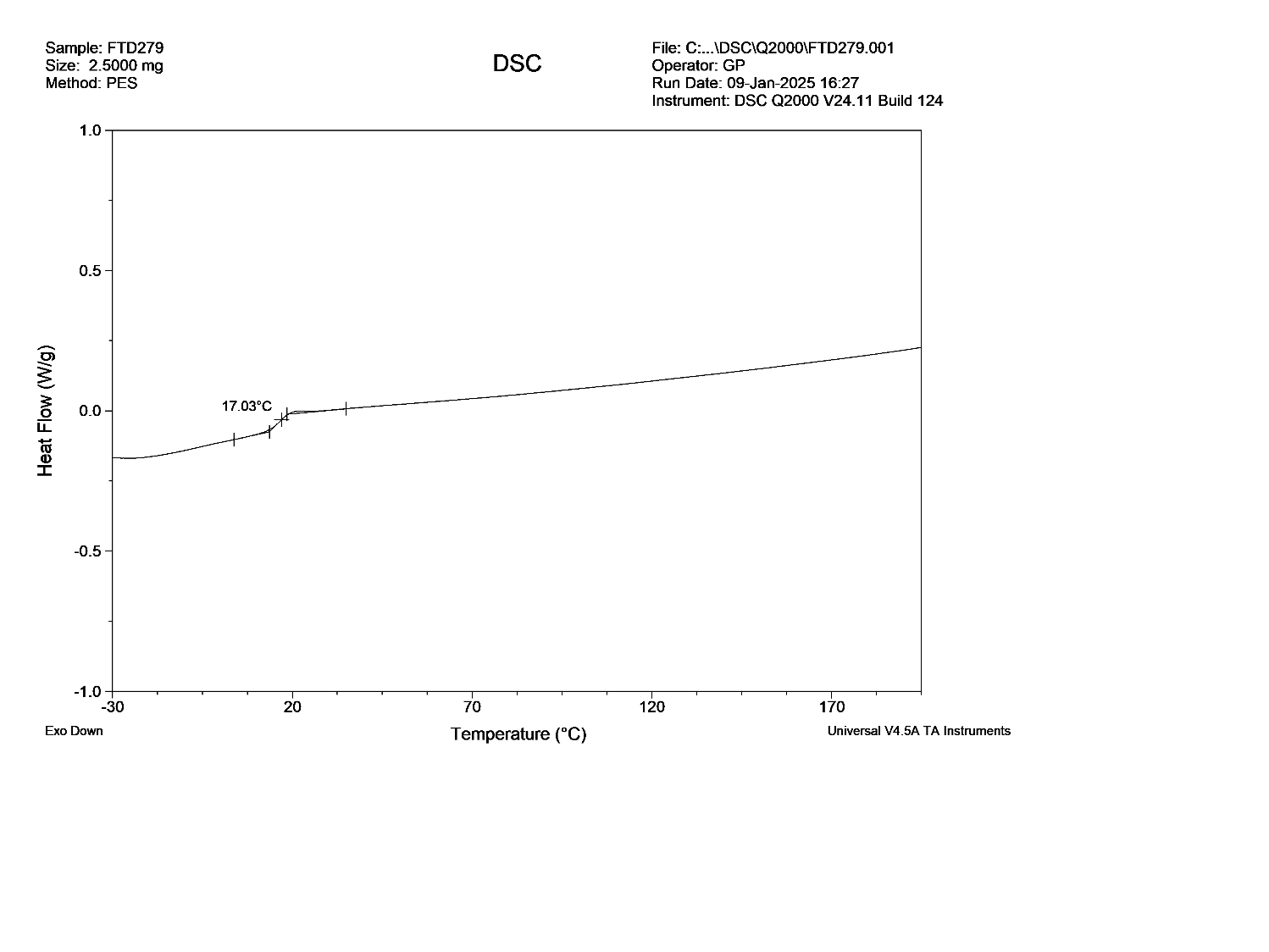


### **Figure S34**: DSC thermogram of PTMC-co-PLA sample (third run). (*entry 3, Table 2*)

14
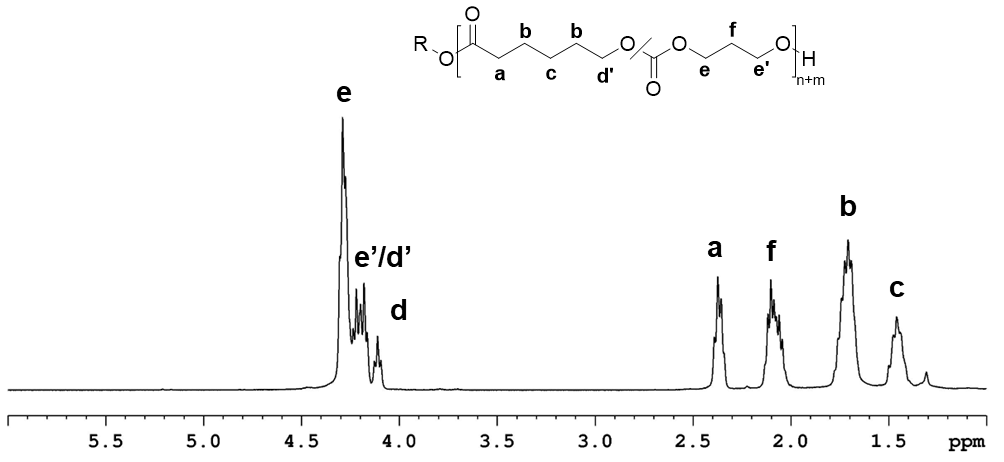


### **Figure S35**: ^1^H NMR spectrum of PTMC-co-PCL (Solvent: CDCl_3_, 400 MHz, 298K). (*entry 6, Table 2)*


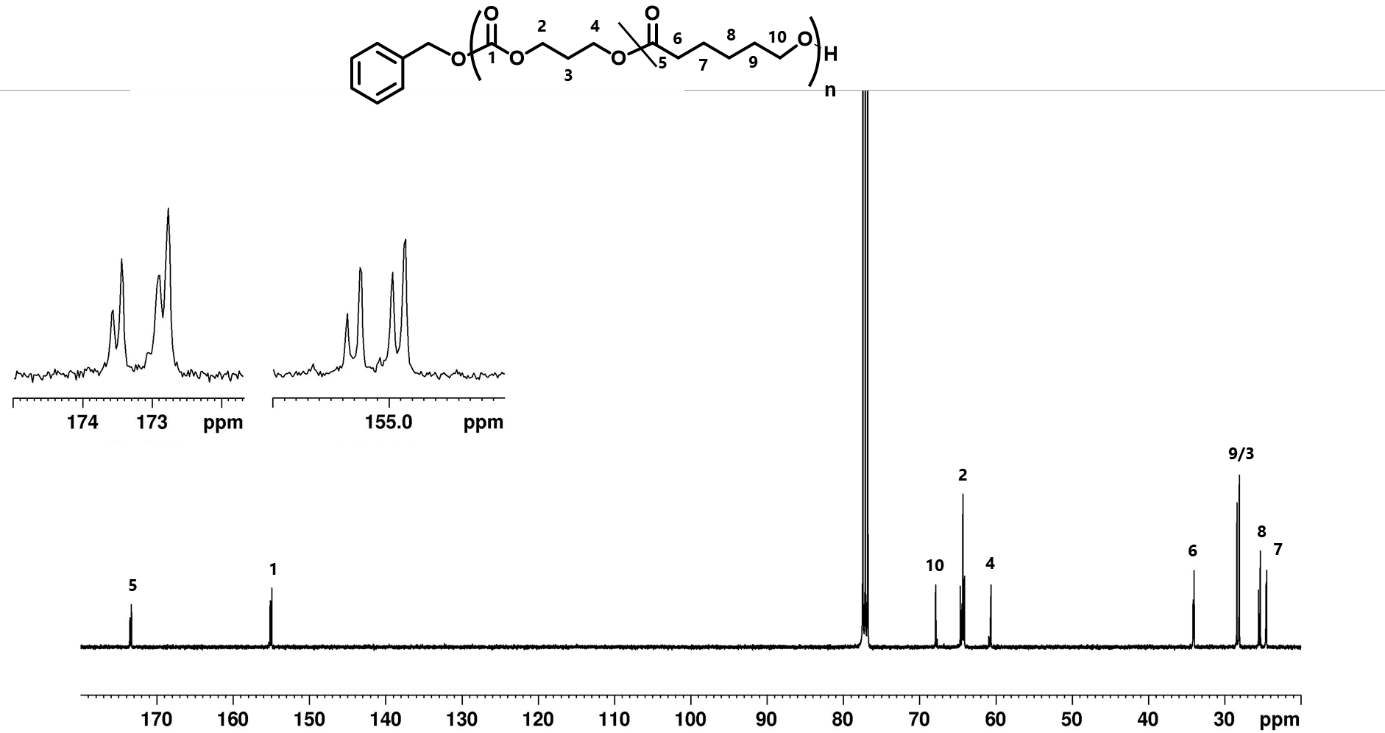


### **Figure S36**: ^13^C NMR spectrum of PTMC-co-PCL (Solvent: CDCl_3_, 100.5 MHz, 298K). (*entry 6, Table 2*)


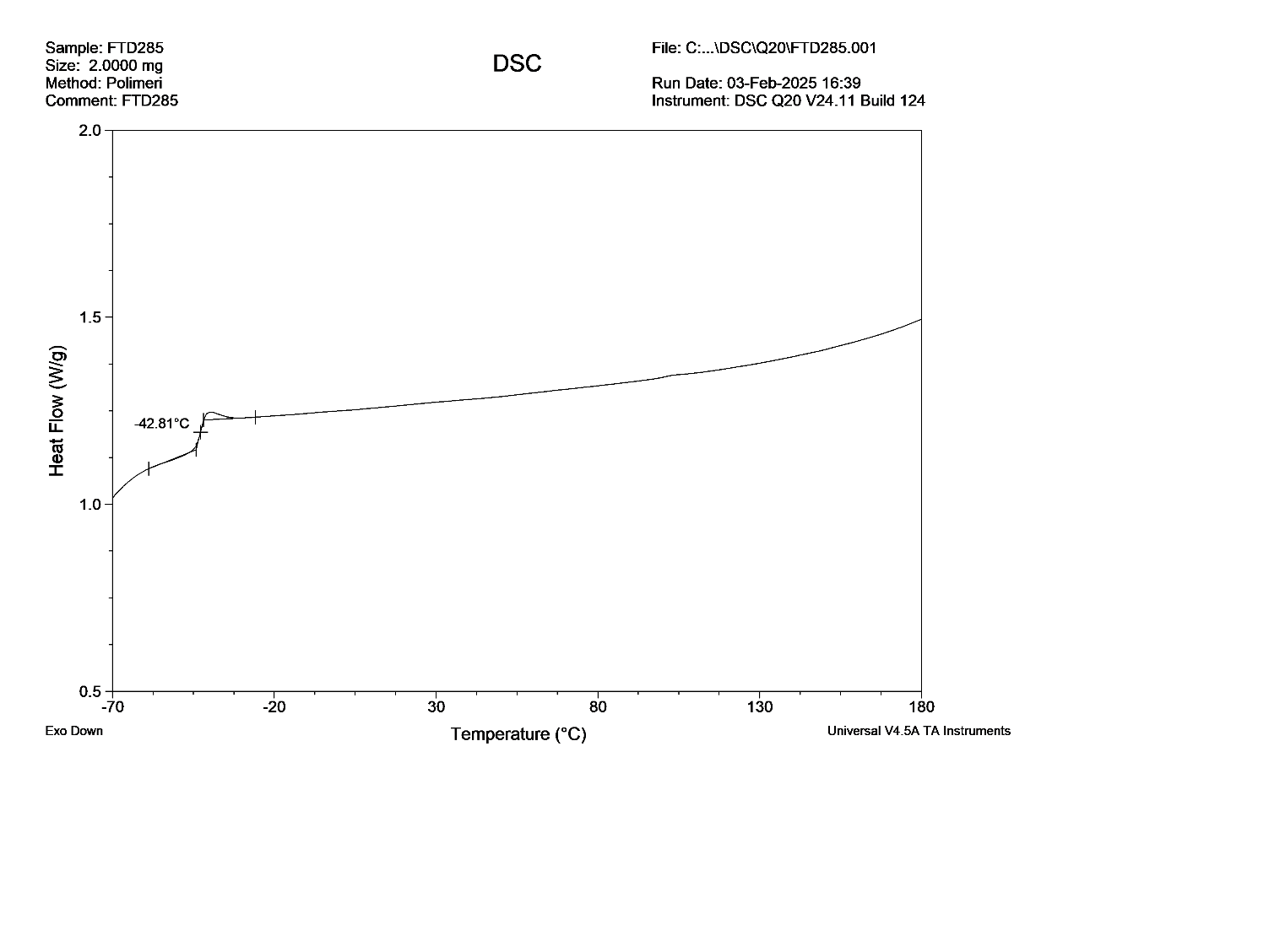


### **Figure S37**: DSC thermogram of PTMC-co-PCL sample (third run). (*entry 5, Table 2*)

# **Chemical recycling of PTMC using microwave irradiation**

Depolymerization experiments were performed: the complex (1.4 × 10^−5^ mol) was weighed into a 4 mL vial and dissolved in 0.4 mL of CH_3_CN. Subsequently, a solution of 1,3-propanediol (1.4 × 10^−5^ mol) in anhydrous acetonitrile was added and left stirring for few minutes. The polymer (12 mg, 10^-4^ mol) was weighted into another vial, dissolved in 0.6 mL of acetonitrile and then placed into microwave vessel. Finally, the reaction mixture was transferred into vessel, closing and taking to a microwave reactor. All the depolymerization experiments were stopped using wet dichloromethane. The suspension was characterized by NMR spectroscopy.


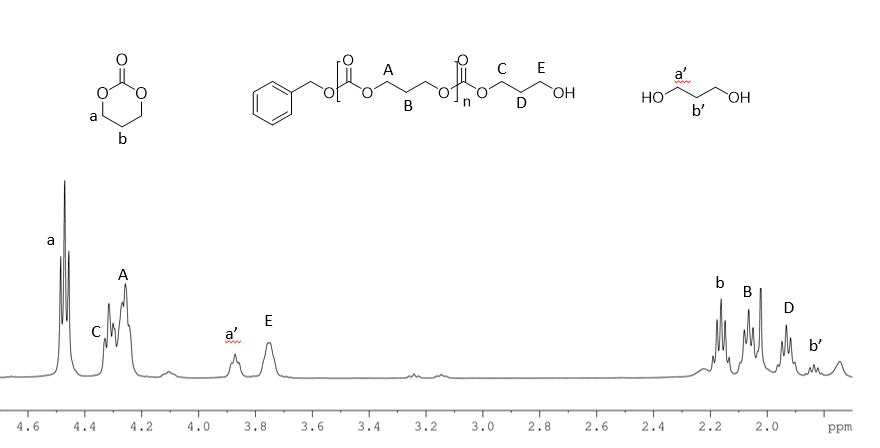


### **Figure S38:** ^1^H NMR spectrum of PTMC depolymerization with assignment of internal alkyl protons, TMC and 1,3 propanediol. (Solvent = CDCl_3_, 400 MHz, 298 K).


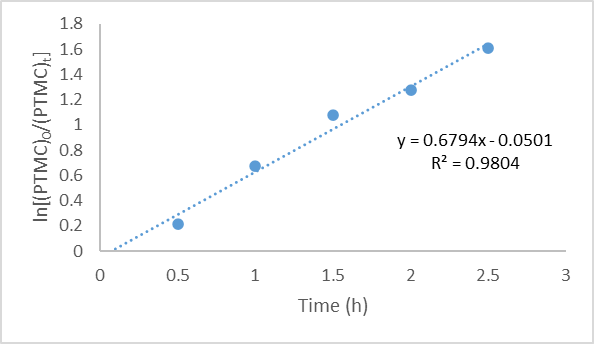


### **Figure S39**: Pseudo first-order kinetic plots for the consumption of PTMC by complex **1** and 1,3 propanediol.

### **Table S3**: Depolymerization of copolymers with complex **1**

| ^a^Entry | ^b^Polymer  (Mn, Ð) | Time | ^c^Conv_TMC_  (%) | ^c^Conv_LLA_  (%) | ^c^Conv_ɛ-CL_  (%) |
| --- | --- | --- | --- | --- | --- |
| 1 | PTMCbPLLA  (10.0 kDa, 1.32) | 0.5 h | 0 | >99 | - |
| 2 | PTMCbPCL  (19.0 kDa, 1.72) | 2 h | 59 | - | 0 |
| 3 | PCL  (10.1 kDa, 1.69) | 2 h | - | - | 0 |

^a^The reactions were performed using 14 µmol of complex **1** (0.15 equiv. with respect to the functional groups in the chain) in 1 mL of CH_3_CN at 95 °C by microwave irradiation. ^b^The polymers used for the depolymerization reactions were synthesized *ad hoc* and washed with methanol to remove all the catalytic residues. The values of *M*_n_ and Ð were determined by SEC in THF. ^c^Determined by ^1^H NMR spectral data.
